# Supplementary material for: Anticholinesterase and Antityrosinase Secondary Metabolites from the Fungus Xylobolus subpileatus
Source: Molecules. 2023 Dec 30;29(1):213. doi: 10.3390/molecules29010213 (PMC10780293; doi:10.3390/molecules29010213)
Supplement: Supplementary file 1 [file molecules-29-00213-s001.zip › molecules-2747628-supplementary.pdf]

Anticholinesterase and Antityrosinase Secondary Metabolites from the Fungus *Xylobolus subpileatus*

Kristóf Felegyi[1], Zsófia Garádi[1,2], Elżbieta Studzińska-Sroka[3], Viktor Papp[4], Imre Boldizsár[1,5], András Dancsó[2], Szabolcs Béni[1,6], Przemysław Zalewski[3] and Attila Ványolós[1,*]

[1] Department of Pharmacognosy, Semmelweis University, 1085 Budapest, Hungary; felegyi.kristof@pharma.semmelweis-univ.hu (K.F.); garadi.zsofia@pharma.semmelweis-univ.hu (Z.G.); boldizsar.imre@semmelweis.hu (I.B.);
[2] Directorate of Drug Substance Development, Egis Pharmaceuticals Plc., 1475 Budapest, Hungary
[3] Department of Pharmacognosy and Biomaterials, Poznan University of Medical Sciences, Rokietnicka 3 Str, 60-806 Poznan, Poland; elastudzinska@ump.edu.pl (E.S.-S.); pzalewski@ump.edu.pl (P.Z.)
[4] Department of Botany, Hungarian University of Agriculture and Life Sciences, 1118 Budapest, Hungary; papp.viktor@uni-mate.hu
[5] Department of Plant Anatomy, Institute of Biology, Eötvös Loránd University, 1117 Budapest, Hungary
[6] Department of Analytical Chemistry, Institute of Chemistry, Eötvös Loránd University, 1117 Budapest, Hungary; szabolcs.beni@ttk.elte.hu

* Correspondence: vanyolos.attila@semmelweis.hu

Contents

Figure S1. HRESI-MS spectrum of compound **1** ... 6

Figure S2. MS-MS spectrum of compound **1** ... 6

Figure S3. ^{1}H spectrum of compound **1** (600 MHz, $CDCl_3$, 295 K) ... 7

Figure S4. ^{13}C spectrum of compound **1** (150 MHz, $CDCL_3$, 295 K) ... 7

Figure S5. COSY spectrum of compound **1** (600 MHz, $CDCl_3$, 295 K) ... 8

Figure S6. DEPT-edited HSQC spectrum of compound **1** (600 MHz, $CDCl_3$, 295 K) ... 8

Figure S7. HMBC spectrum of compound **1** (600 MHz, $CDCl_3$, 295 K) ... 9

Figure S8. ROESY spectrum of compound **1** (600 MHz, $CDCl_3$, 295 K) ... 9

Figure S9. HRESI-MS spectrum of compound **2** ... 10

Figure S10. MS-MS spectrum of compound **2** ... 10

Figure S11. ^{1}H spectrum of compound **2** (600 MHz, $CDCl_3$, 295 K) ... 11

Figure S12. ^{13}C spectrum of compound **2** (150 MHz, $CDCl_3$, 295 K) ... 11

Figure S13. COSY spectrum of compound **2** (600 MHz, $CDCl_3$, 295 K) ... 12

Figure S14. DEPT-edited HSQC spectrum of compound **2** (600 MHz, $CDCl_3$, 295 K) ... 12

Figure S15. HMBC spectrum of compound **2** (600 MHz, $CDCl_3$, 295 K) ... 13

Figure S16. ROESY spectrum of compound **2** (600 MHz, $CDCl_3$, 295 K) ... 13

Figure S17. HRESI-MS spectrum of compound **3** ... 14

Figure S18. MS-MS spectrum of compound **3** ... 14

Figure S19. ^{1}H spectrum of compound **3** (600 MHz, $CDCl_3$, 295 K) ... 15

Figure S20. ^{13}C spectrum of compound **3** (150 MHz, $CDCl_3$, 295 K) ... 15

Figure S21. COSY spectrum of compound **3** (600 MHz, $CDCl_3$, 295 K) ... 16

Figure S22. DEPT-edited HSQC spectrum of compound **3** (600 MHz, $CDCl_3$, 295 K) ... 16

Figure S23. HMBC spectrum of compound **3** (600 MHz, $CDCl_3$, 295 K ... 17

Figure S24. HRESI-MS spectrum of compund **4** ... 18

Figure S25. MS-MS spectrum of compound 4 ... 18

Figure S26. ^{1}H spectrum of compound **4** (600 MHz, THF-d_8, 295 K) ... 19

Figure S27. ^{13}C spectrum of compound **4** (150 MHz, THF-d_8, 295 K) ... 19

Figure S28. COSY spectrum of compound **4** (600 MHz, THF-d_8, 295 K) ... 20

Figure S29. DEPT-edited HSQC spectrum of compound **4** (600 MHz, THF-d_8, 295 K) ... 20

Figure S30. HMBC spectrum of compound **4** (600 MHz, THF-d_8, 295 K) ... 21

Figure S31. ROESY spectrum of compound **4** (600 MHz, THF-d_8, 295 K) ... 21

Figure S32. ^{1}H spectrum of compound **5** (600 MHz, $CDCl_3$, 295 K) ... 22

Figure S33. ^{13}C spectrum of compound **5** (150 MHz, $CDCl_3$, 295 K) 23
Figure S34. COSY spectrum of compound **5** (600 MHz, $CDCl_3$, 295 K) 23
Figure S35. DEPT-edited HSQC spectrum of compound **5** (600 MHz, $CDCl_3$, 295 K) 24
Figure S36. HMBC spectrum of compound **5** (600 MHz, $CDCl_3$, 295 K) 24
Figure S37. ROESY spectrum of compound **5** (600 MHz, $CDCl_3$, 295 K) 25
Figure S38. HRESI-MS spectrum of compound **6** 26
Figure S39. MS-MS spectrum of compound **6** 26
Figure S40. ^{1}H spectrum of compound **6** (600 MHz, THF-d_8, 295 K) 27
Figure S41. ^{13}C spectrum of compound **6** (150 MHz, THF-d_8, 295 K) 27
Figure S42. COSY spectrum of compound **6** (600 MHz, THF-d_8, 295 K) 28
Figure S43. DEPT-edited HSQC spectrum of compound **6** (600 MHz, THF-d_8, 295 K) 28
Figure S44. HMBC spectrum of compound **6** (600 MHz, THF-d_8, 295 K) 29
Figure S45. ROESY spectrum of compound **6** (600 MHz, THF-d_8, 295 K) 29
Figure S46. HRESI-MS spectrum of compound **7** 30
Figure S47. MS-MS spectrum of compound **7** 30
Figure S48. ^{1}H spectrum of compound **7** (600 MHz, THF-d_8, 295 K) 31
Figure S49. ^{13}C spectrum of compound **7** (150 MHz, THF-d_8, 295 K) 31
Figure S50. COSY spectrum of compound **7** (600 MHz, THF-d_8, 295 K) 32
Figure S51. DEPT-edited HSQC spectrum of compound **7** (600 MHz, THF-d_8, 295 K) 32
Figure S52. HMBC spectrum of compound **7** (600 MHz, THF-d_8, 295 K) 33
Figure S53. ROESY spectrum of compound **7** (600 MHz, THF-d_8, 295 K) 33
Table S1. Complete ^{1}H and ^{13}C NMR resonance assignments for compounds **1** and **2**. 34
Table S2. Complete ^{1}H and ^{13}C NMR resonance assignments for compounds **4**,**5**,**6** and **7**. 35
Table S3. Complete ^{1}H and ^{13}C NMR resonance assignments for compound **3** 37

Spectra and spectral data on compound **1**

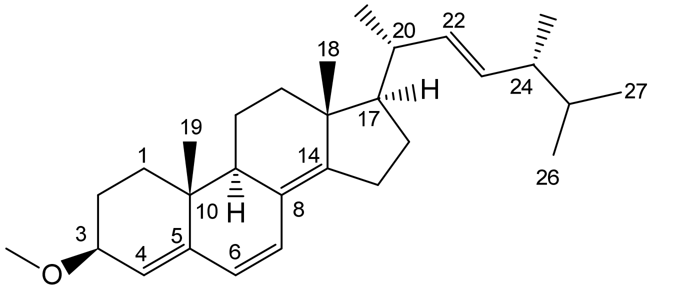

3β-methoxy-ergosta-6,8^{14},22-trien (1): amorphous solid; α_D^{25}+87.8 (*c* 0.42, $CHCl_3$, 25.4 °C); HRESIMS m/z 377,31924 $[M + H - CH_3OH]^+$ (Δ 2.2 ppm; $C_{28}H_{41}$); HRESI-MSMS (CID = 15%, 30%, 45%; rel int %) m/z 293 (16), 251 (24).

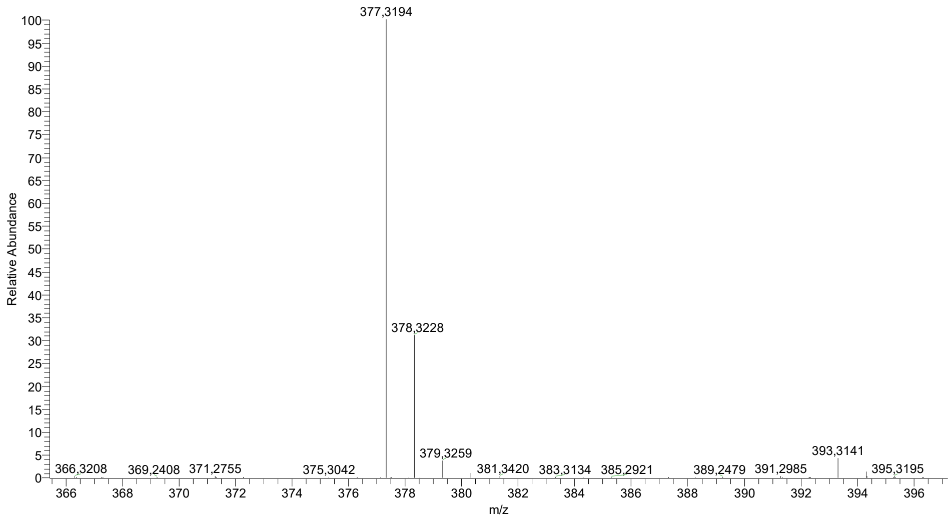

Figure S1. HRESI-MS spectrum of compound **1**

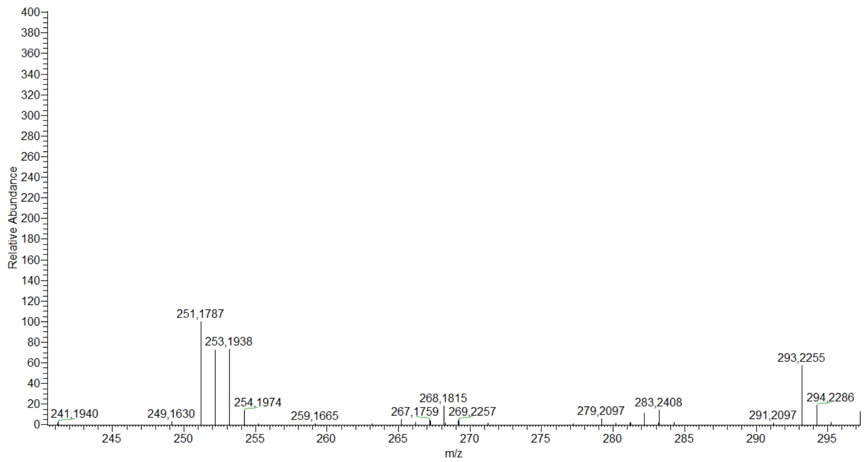

Figure S2. MS-MS spectrum of compound **1**

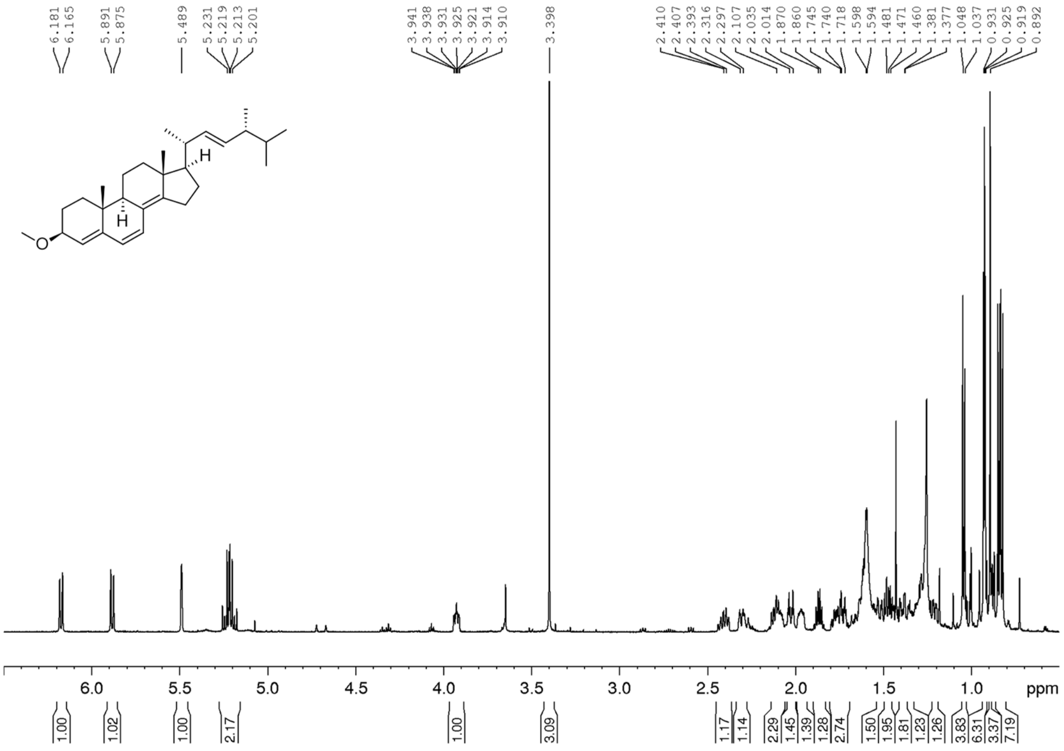

Figure S1. ^{1}H spectrum of compound **1** (600 MHz, $CDCl_3$, 295 K)

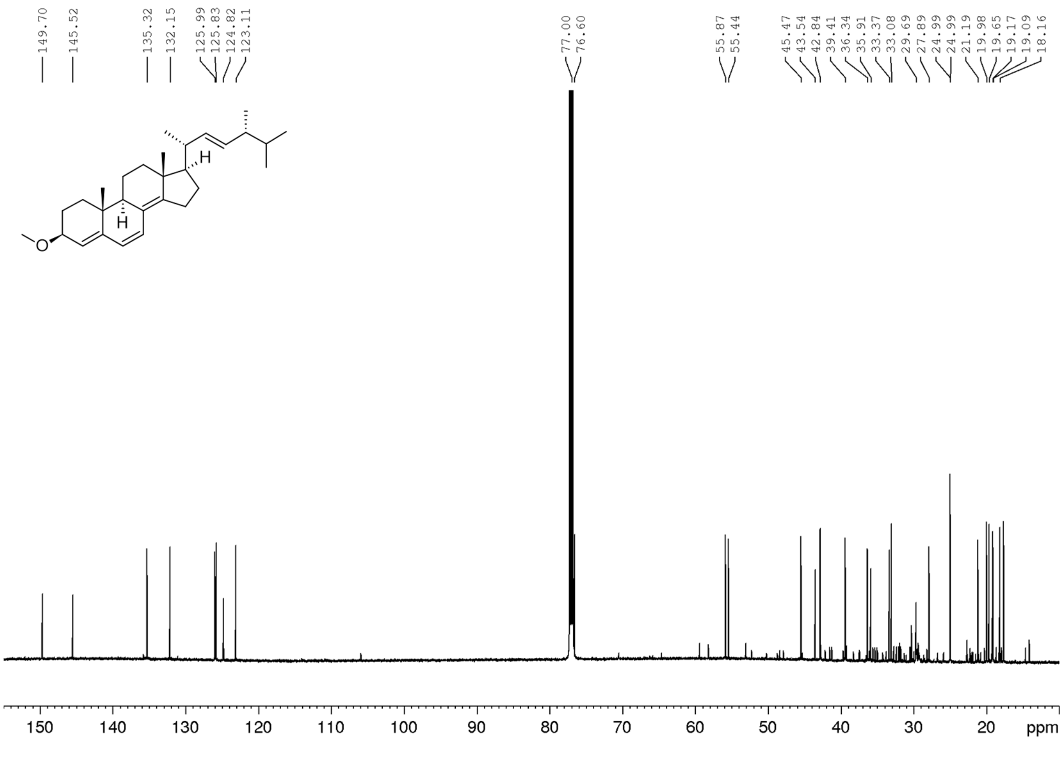

Figure S2. ^{13}C spectrum of compound **1** (150 MHz, $CDCl_3$, 295 K)

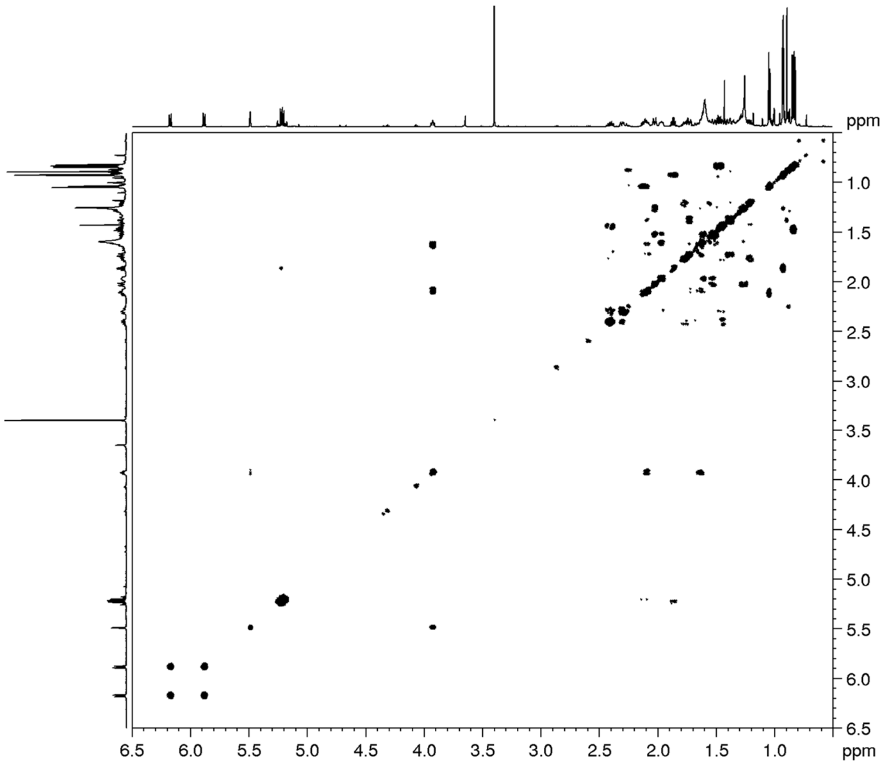

Figure S3. COSY spectrum of compound **1** (600 MHz, $CDCl_3$, 295 K)

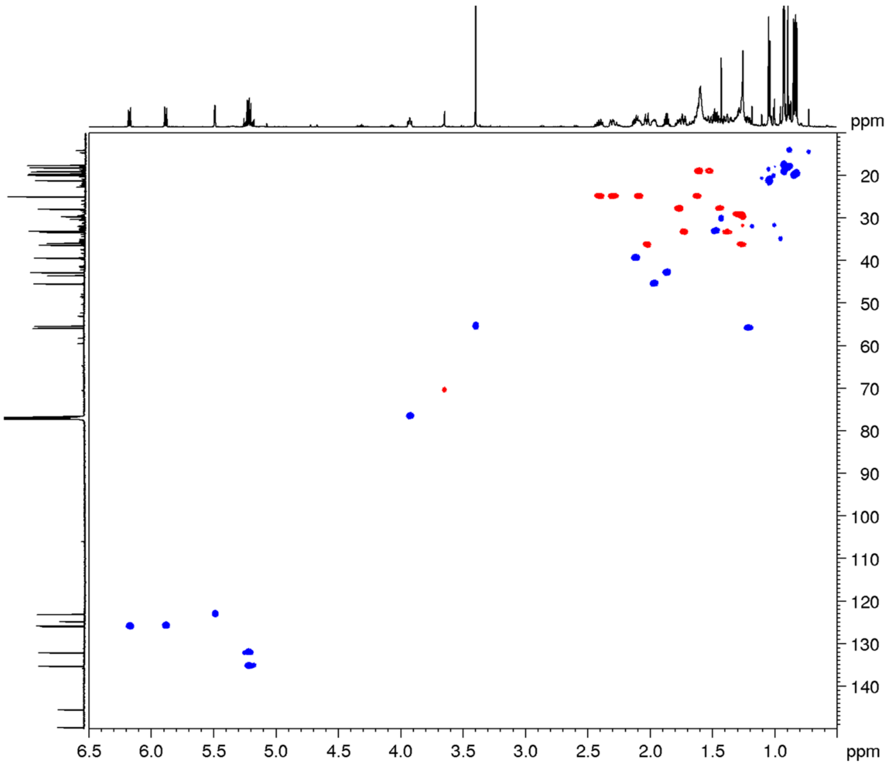

Figure S4. DEPT-edited HSQC spectrum of compound **1** (600 MHz, $CDCl_3$, 295 K)

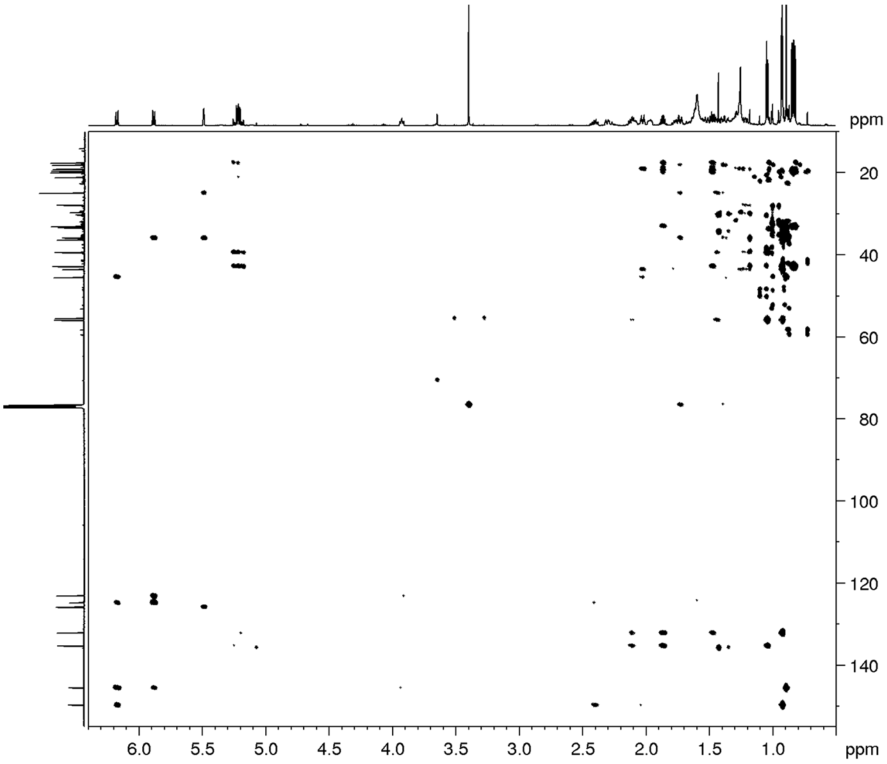

Figure S5. HMBC spectrum of compound **1** (600 MHz, $CDCl_3$, 295 K)

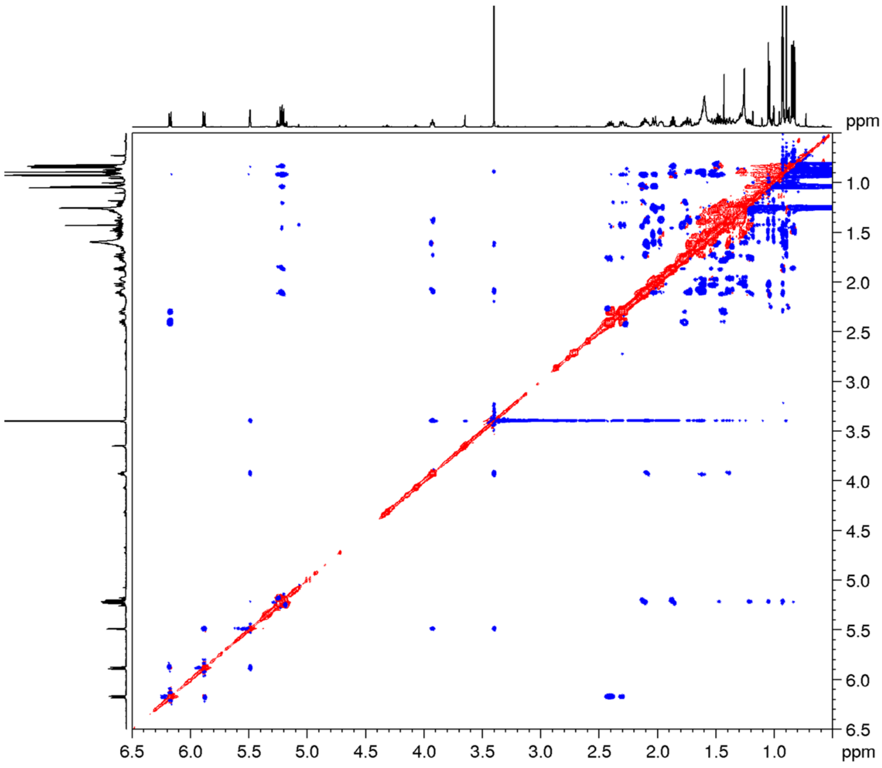

Figure S6. ROESY spectrum of compound **1** (600 MHz, $CDCl_3$, 295 K)

Spectra and spectral data on compound **2**

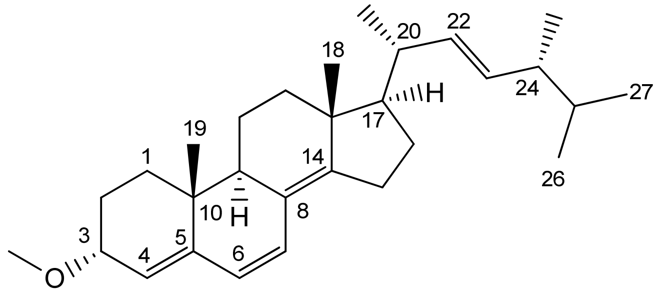

3α-methoxy-ergosta-6,8^{14},22-trien (2): amorphous solid; α_D^{25} -10.0 (*c* 0.09, $CHCl_3$, 25.5 °C); HRESIMS m/z 377,31943 $[M + H - CH_3OH]^+$ (Δ 2.2 ppm; $C_{28}H_{41}$); HRESI-MSMS (CID = 15%, 30%, 45%; rel int %) m/z .293, 251.

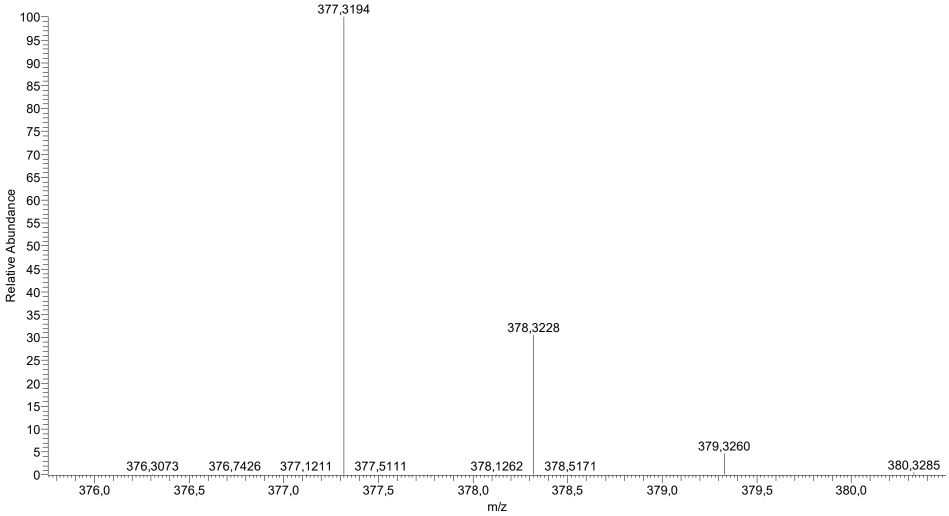

Figure S9. HRESI-MS spectrum of compound **2**

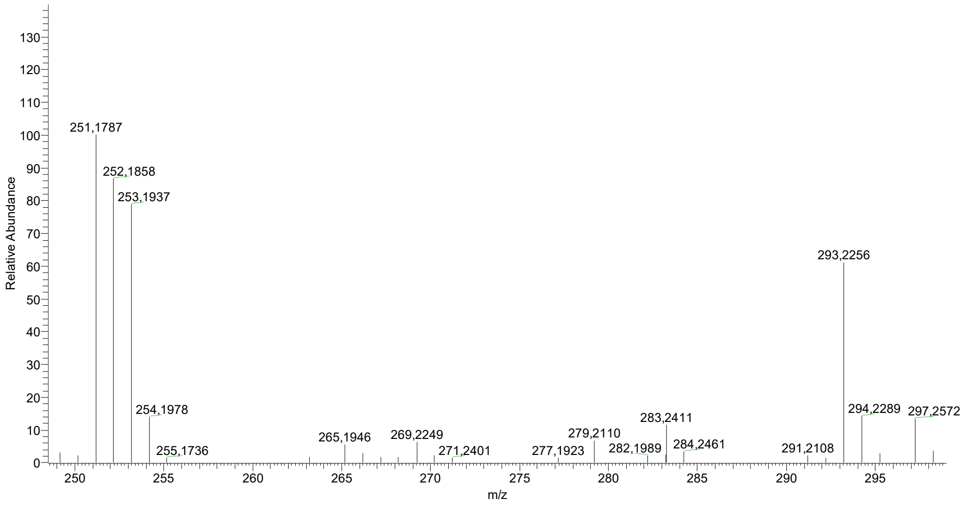

Figure S10. MS-MS spectrum of compound **2**

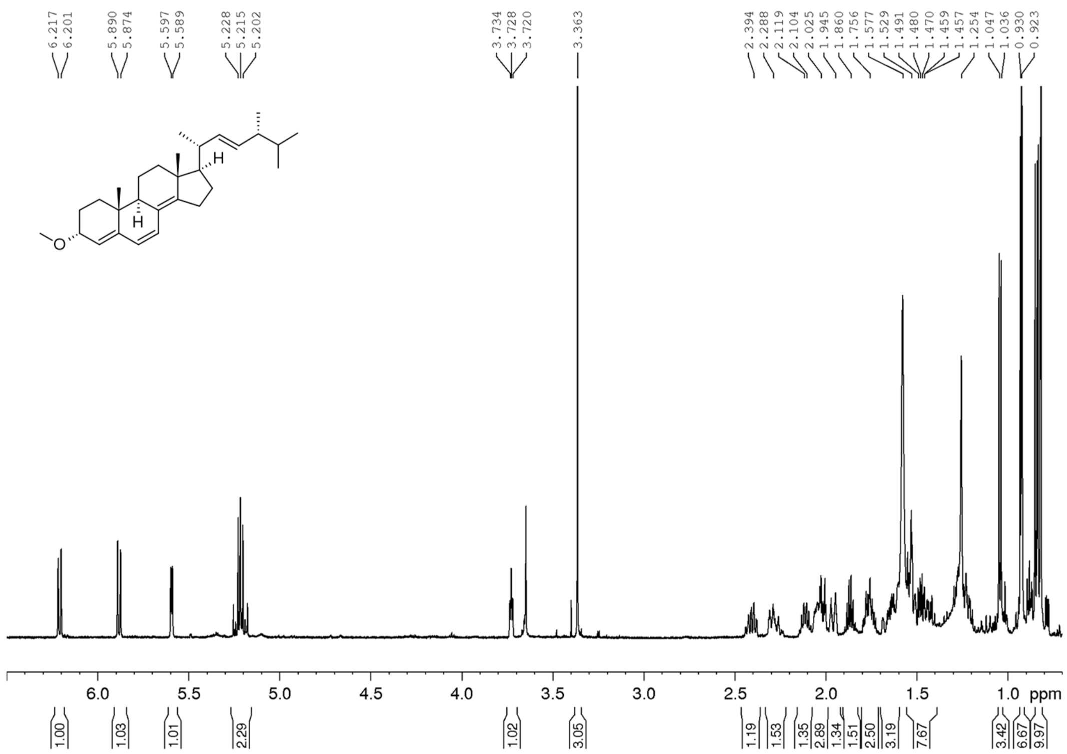

Figure S7. ^{1}H spectrum of compound **2** (600 MHz, $CDCl_3$, 295 K)

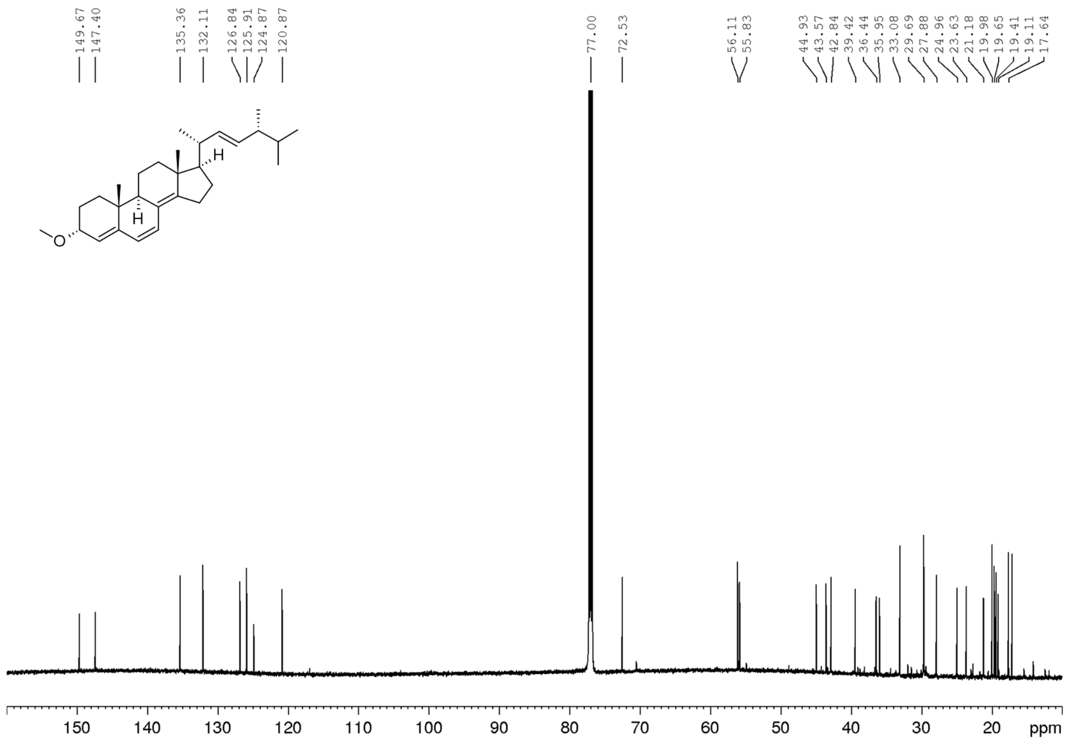

Figure S8. ^{13}C spectrum of compound **2** (150 MHz, $CDCl_3$, 295 K)

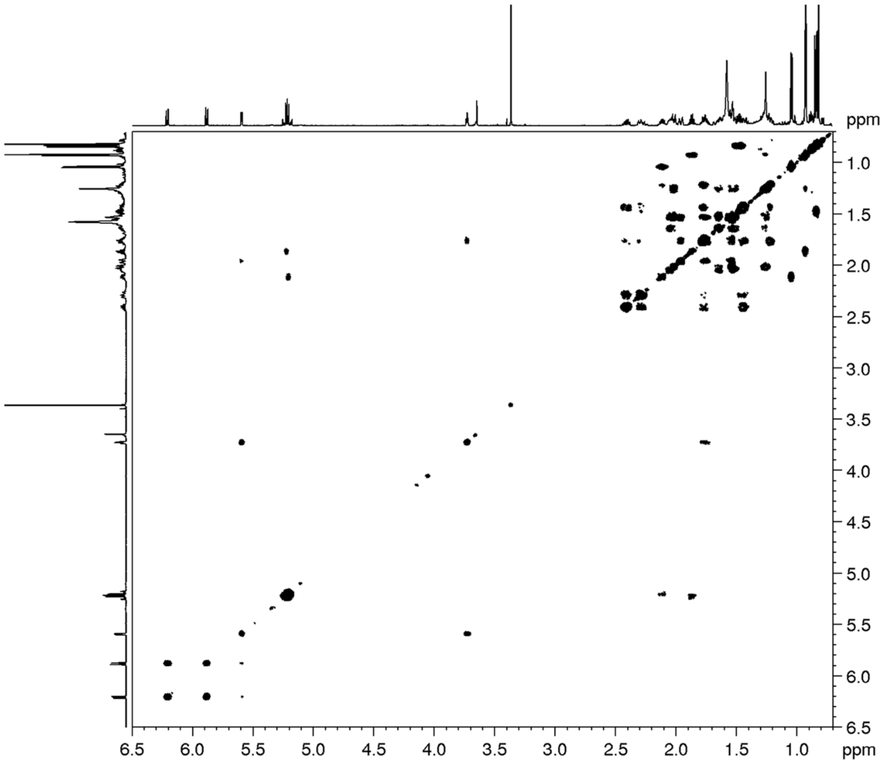

Figure S9. COSY spectrum of compound **2** (600 MHz, $CDCl_3$, 295 K)

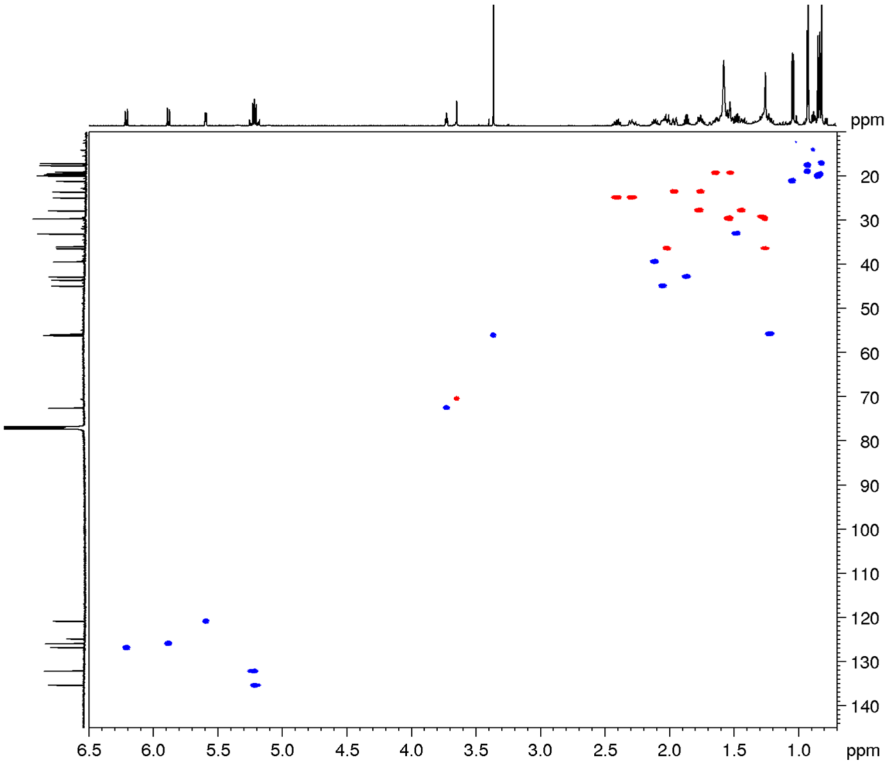

Figure S10. DEPT-edited HSQC spectrum of compound **2** (600 MHz, $CDCl_3$, 295 K)

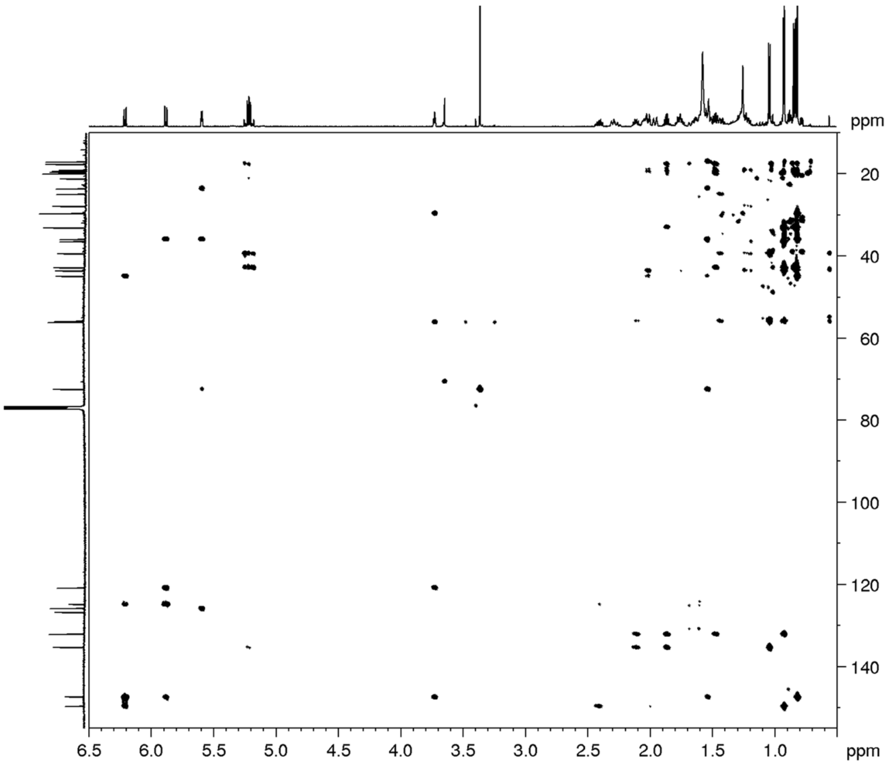

Figure S11. HMBC spectrum of compound **2** (600 MHz, $CDCl_3$, 295 K)

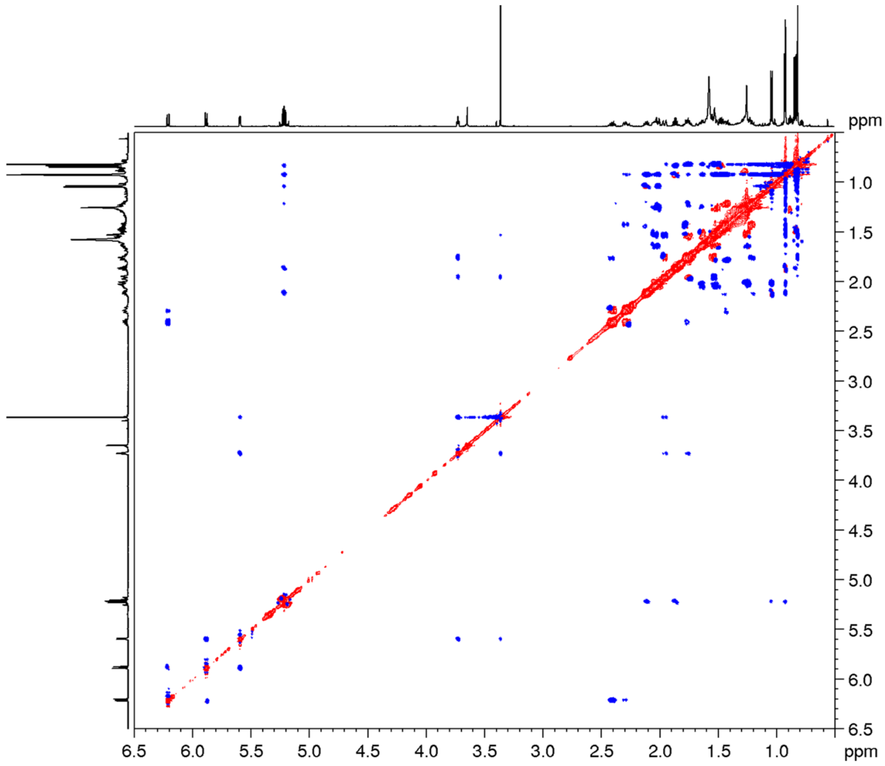

Figure S12. ROESY spectrum of compound **2** (600 MHz, $CDCl_3$, 295 K)

Spectra and spectral data on compound **3**

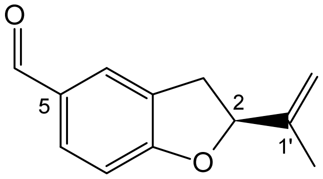

Fomannoxin (3): amorphous solid; HRESIMS m/z 189,09061 [M + H]$^{+}$ (Δ 2.1 ppm; $C_{12}H_{13}O_2$); HRESI-MSMS (CID = 15%, 30%, 45%; rel int %) m/z 161 (97), 143 (23), 133 (33).

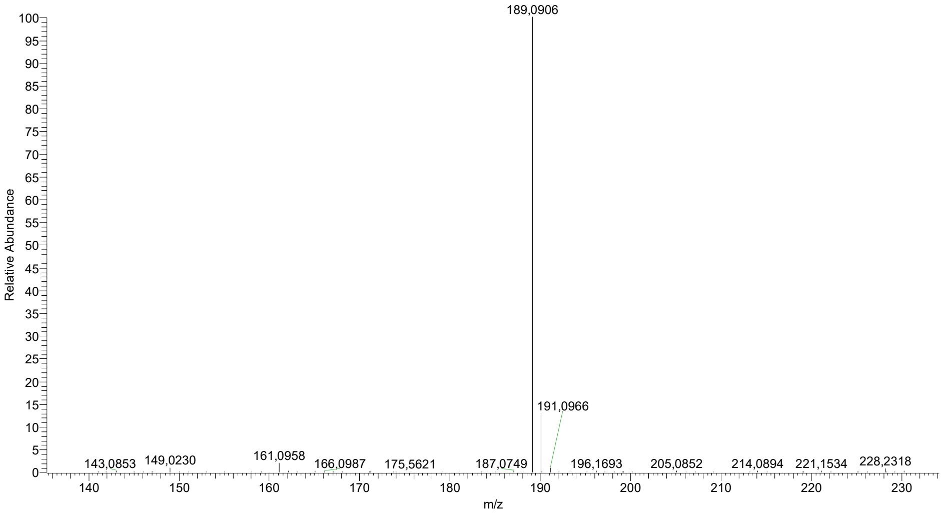

Figure S13. HRESI-MS spectrum of compound **3**

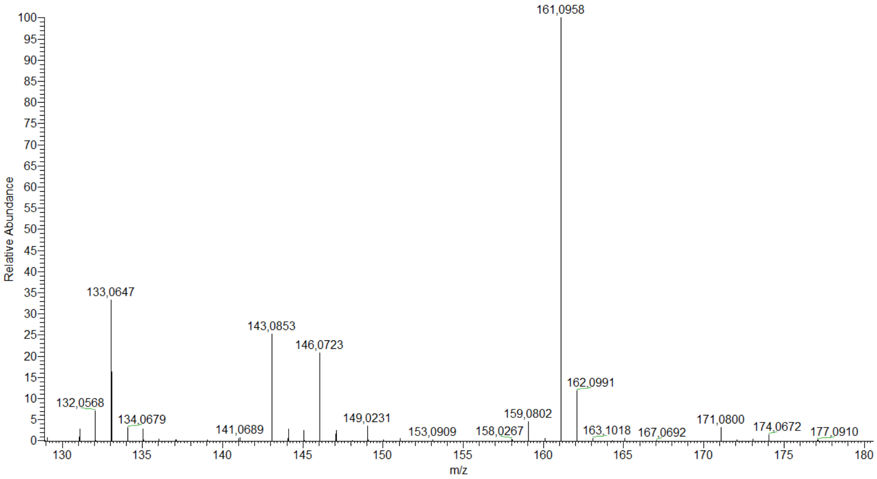

Figure S14. MS-MS spectrum of compound **3**

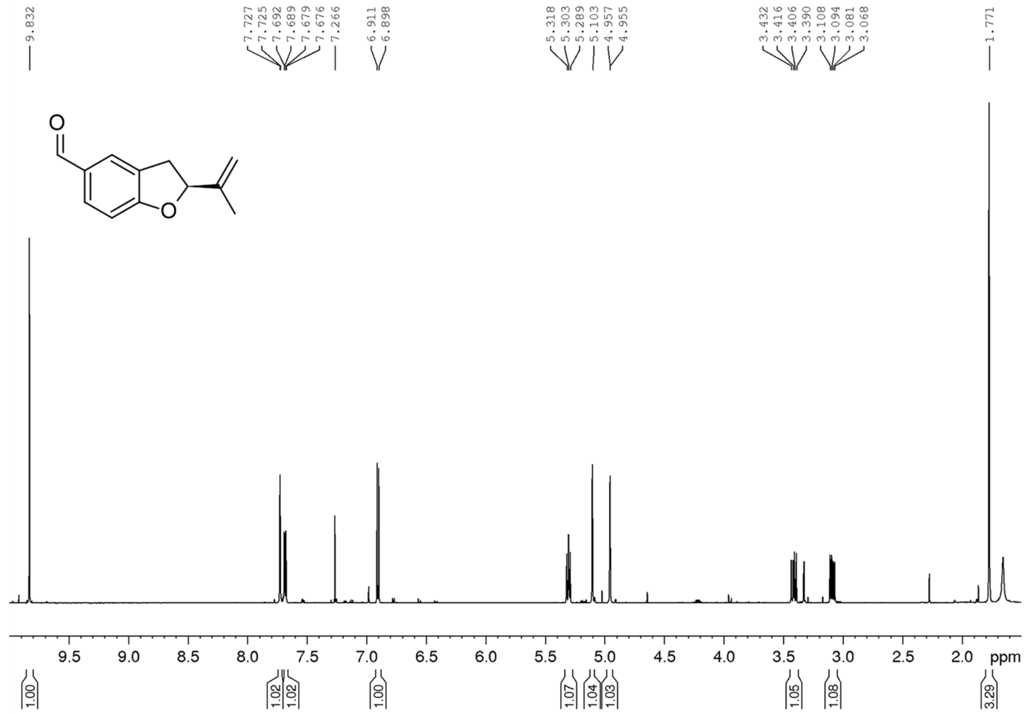

Figure S15. ^{1}H spectrum of compound **3** (600 MHz, $CDCl_3$, 295 K)

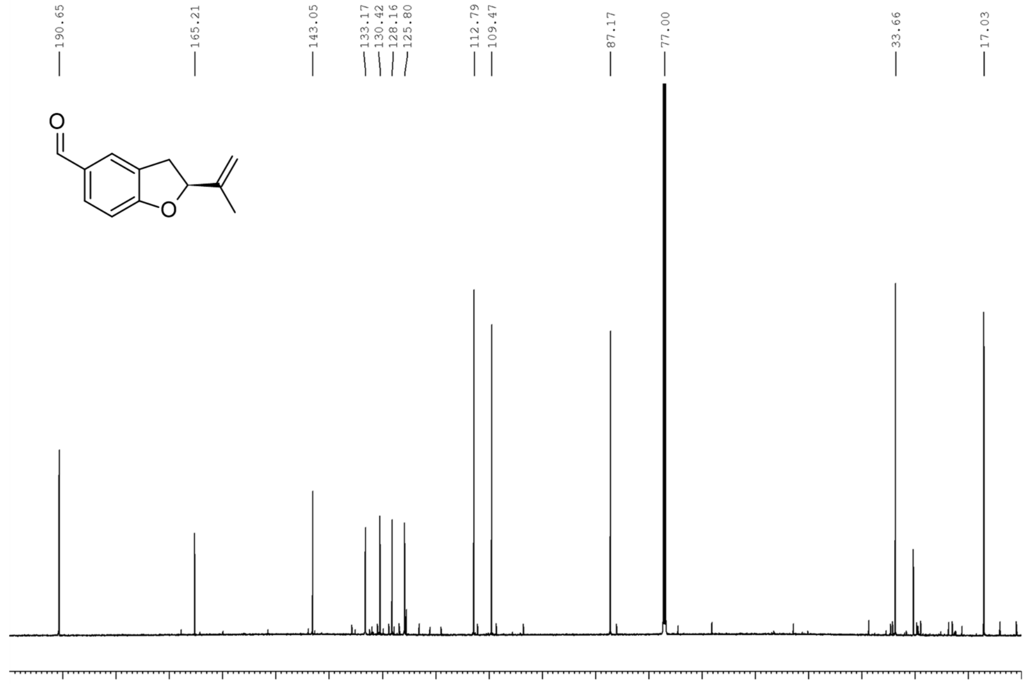

Figure S16. ^{13}C spectrum of compound **3** (150 MHz, $CDCl_3$, 295 K)

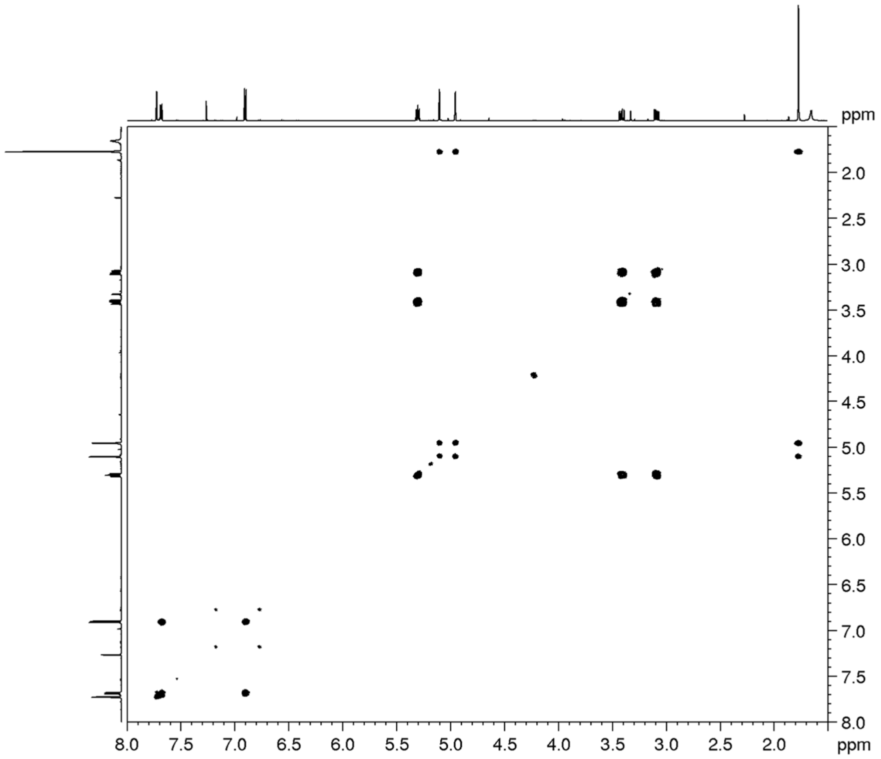

Figure S17. COSY spectrum of compound **3** (600 MHz, $CDCl_3$, 295 K)

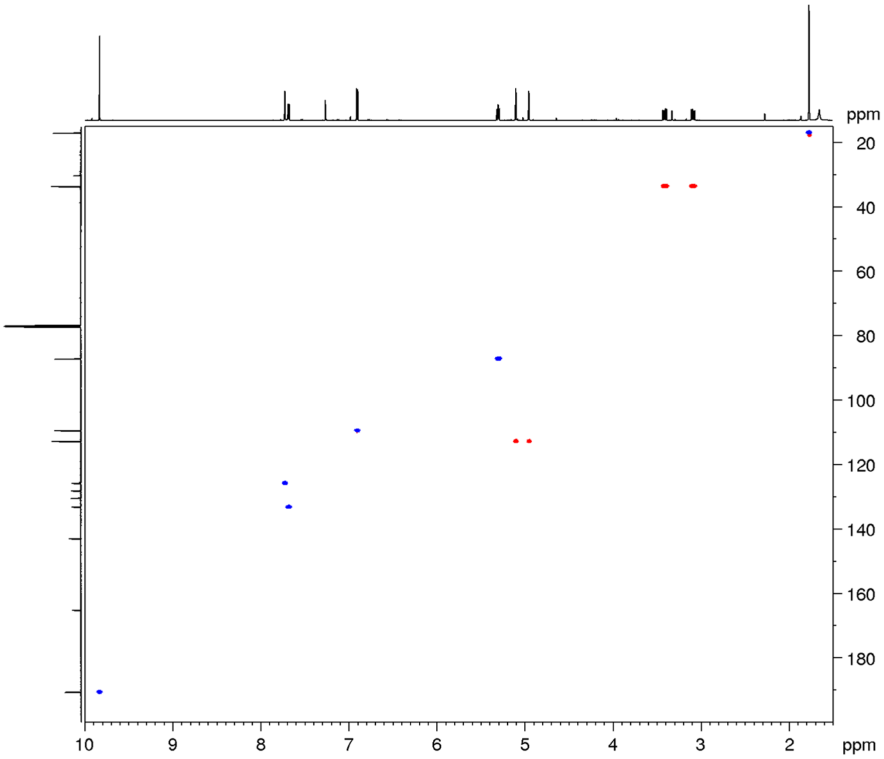

Figure S18. DEPT-edited HSQC spectrum of compound **3** (600 MHz, $CDCl_3$, 295 K)

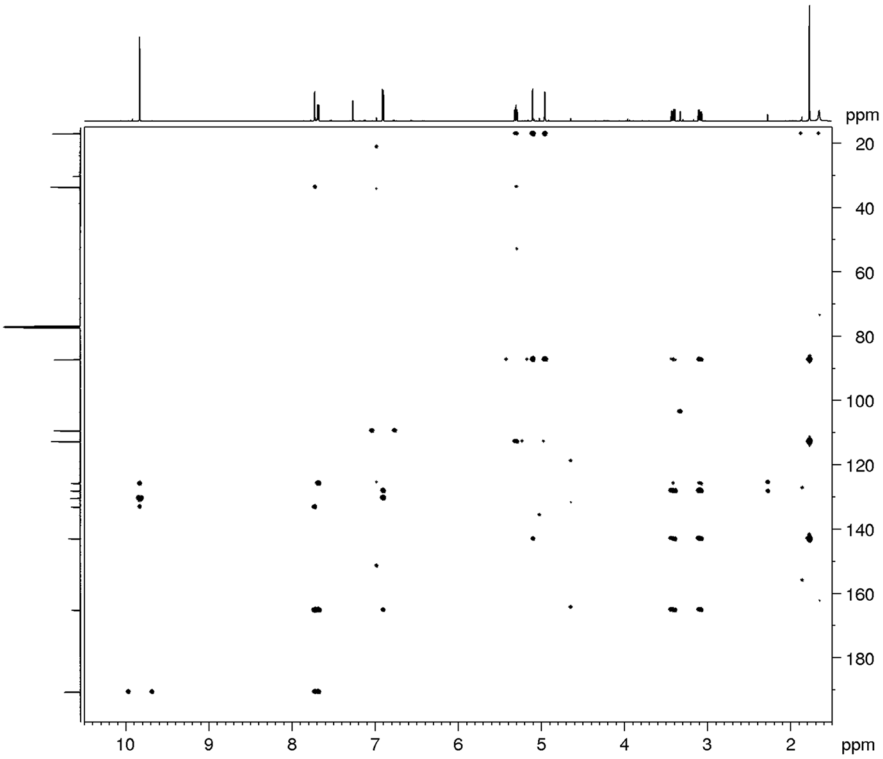

Figure S19. HMBC spectrum of compound **3** (600 MHz, $CDCl_3$, 295 K

Spectra and spectral data on compound **4**

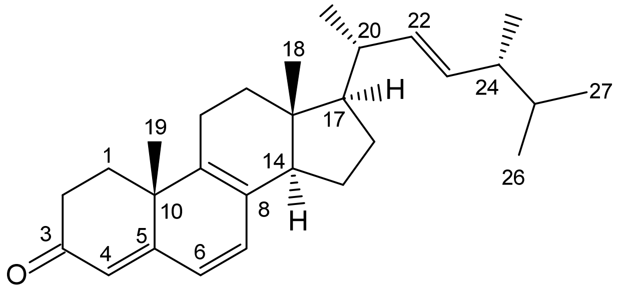

Ergosta-4,6,8[9]22-tetraene-3-one (4): amorphous solid; HRESIMS m/z 393.31458 $[M + H]^+$ (Δ 1.6 ppm; $C_{28}H_{41}O$); HRESI-MSMS (CID = 15%, 30%, 45%; rel int %) m/z 268 (54).

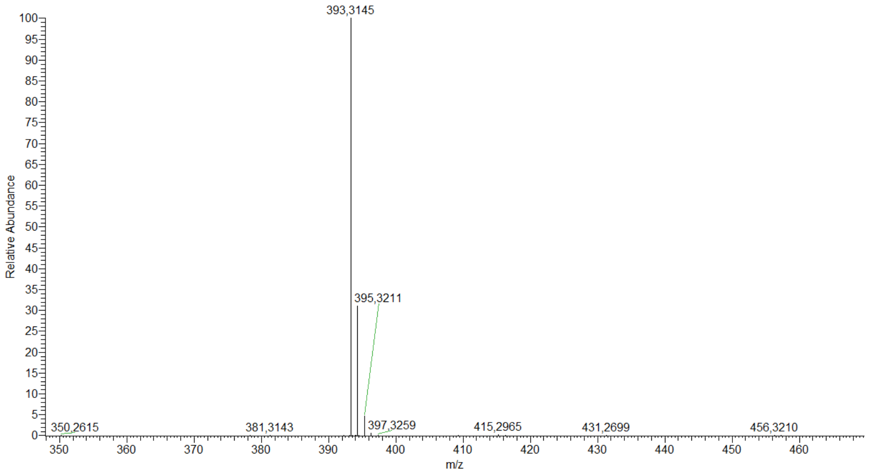

Figure S20. HRESI-MS spectrum of compound **4**

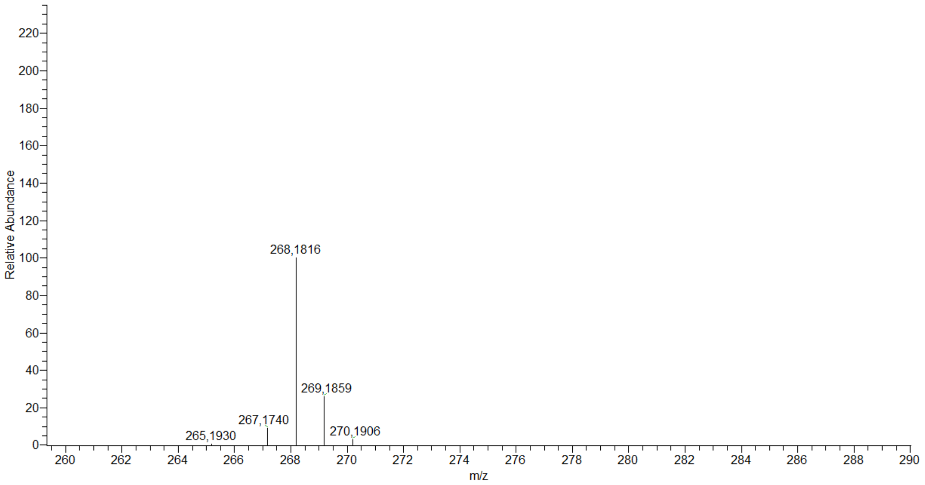

Figure S21. MS-MS spectrum of compound 4

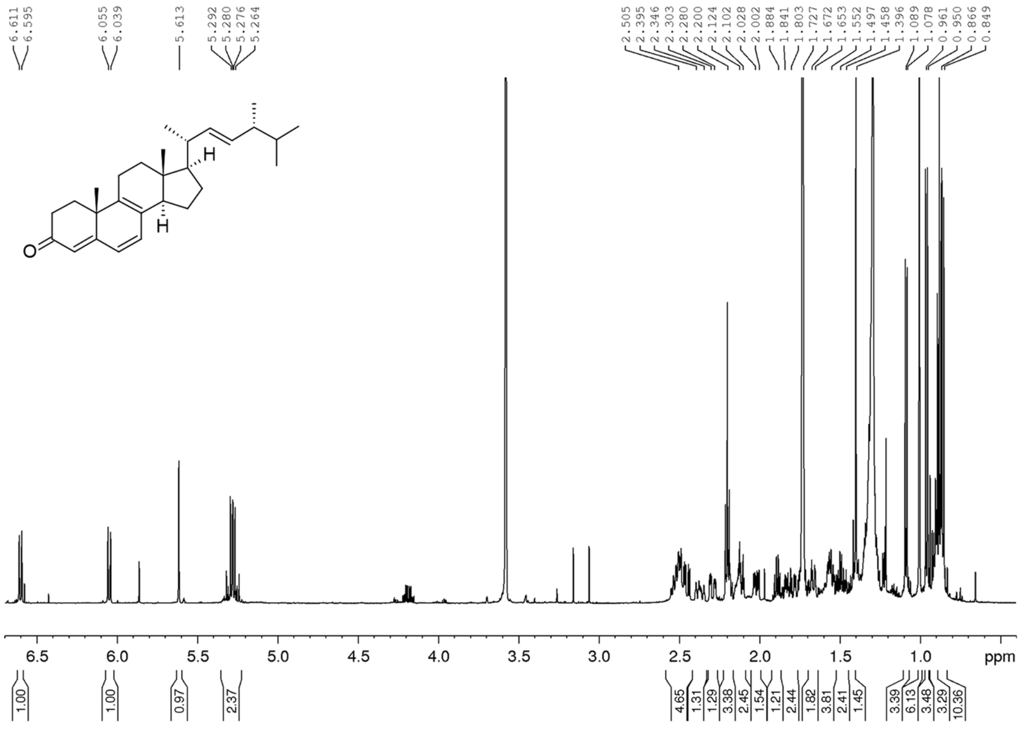

Figure S22. ^{1}H spectrum of compound **4** (600 MHz, THF-d_8, 295 K)

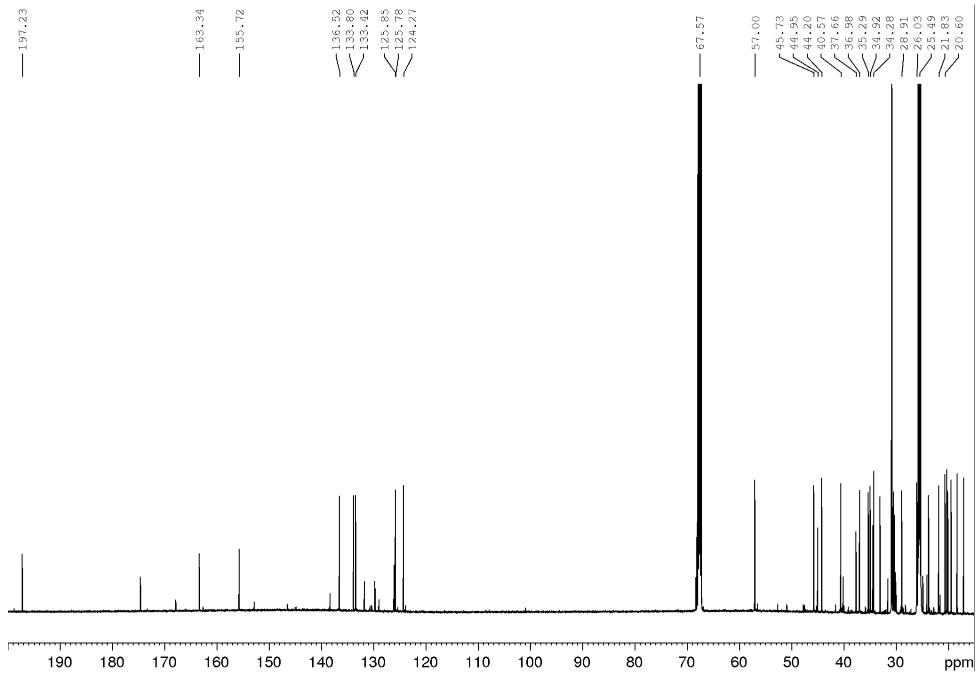

Figure S23. ^{13}C spectrum of compound **4** (150 MHz, THF-d_8, 295 K)

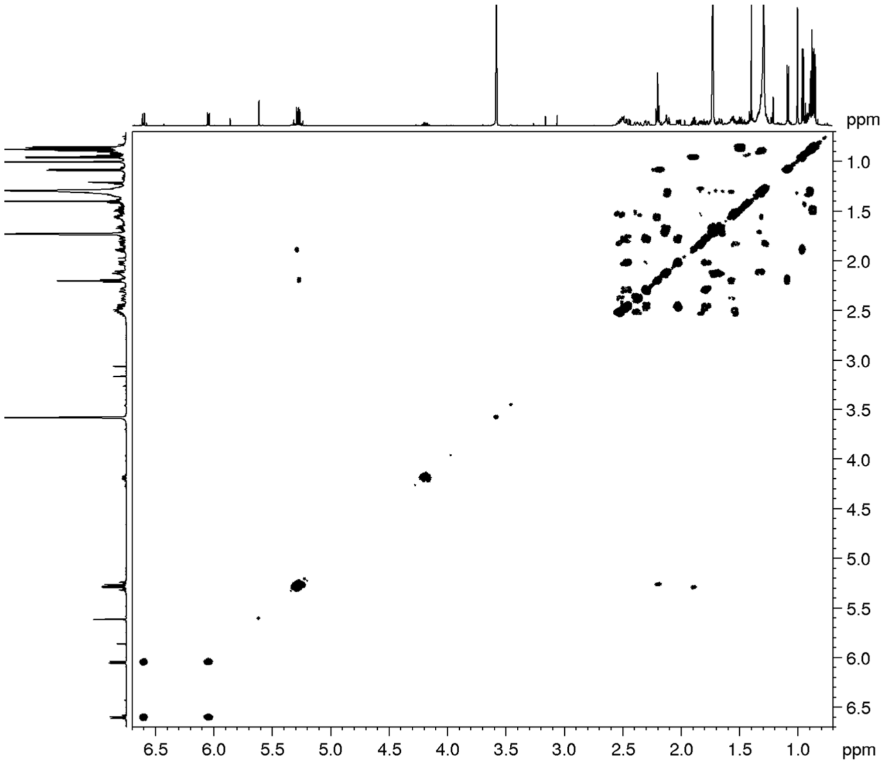

Figure S24. COSY spectrum of compound **4** (600 MHz, THF-d_8, 295 K)

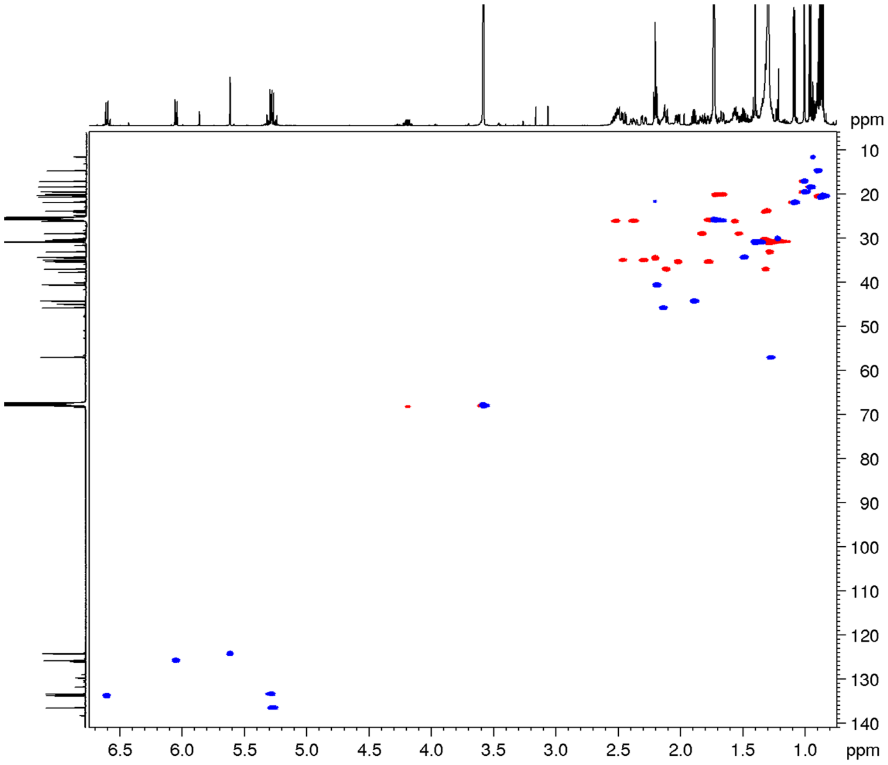

Figure S25. DEPT-edited HSQC spectrum of compound **4** (600 MHz, THF-d_8, 295 K)

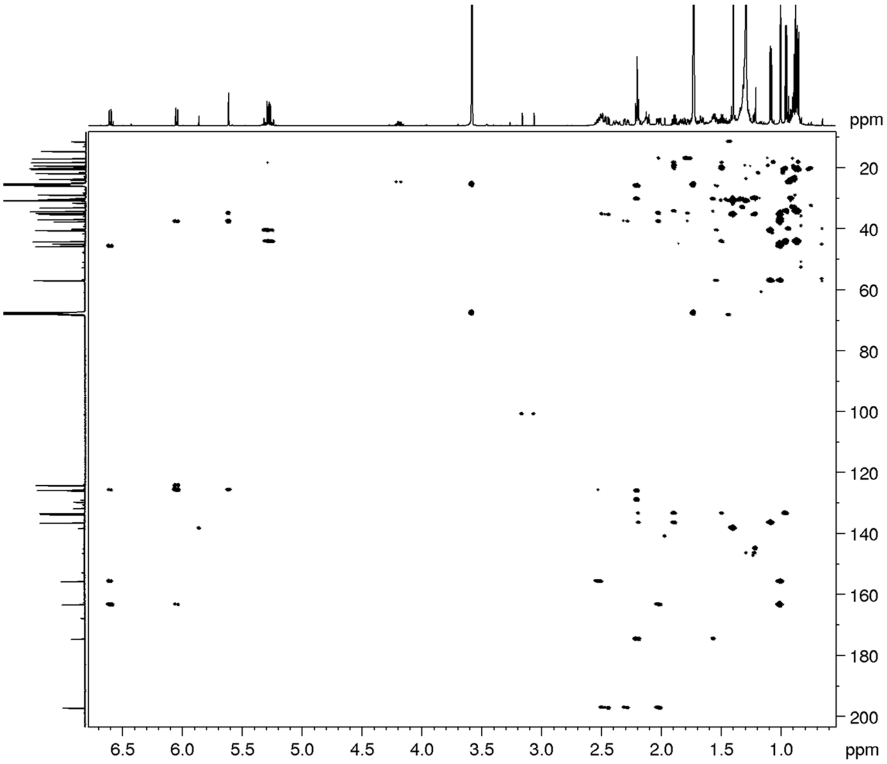

Figure S26. HMBC spectrum of compound **4** (600 MHz, THF-d_8, 295 K)

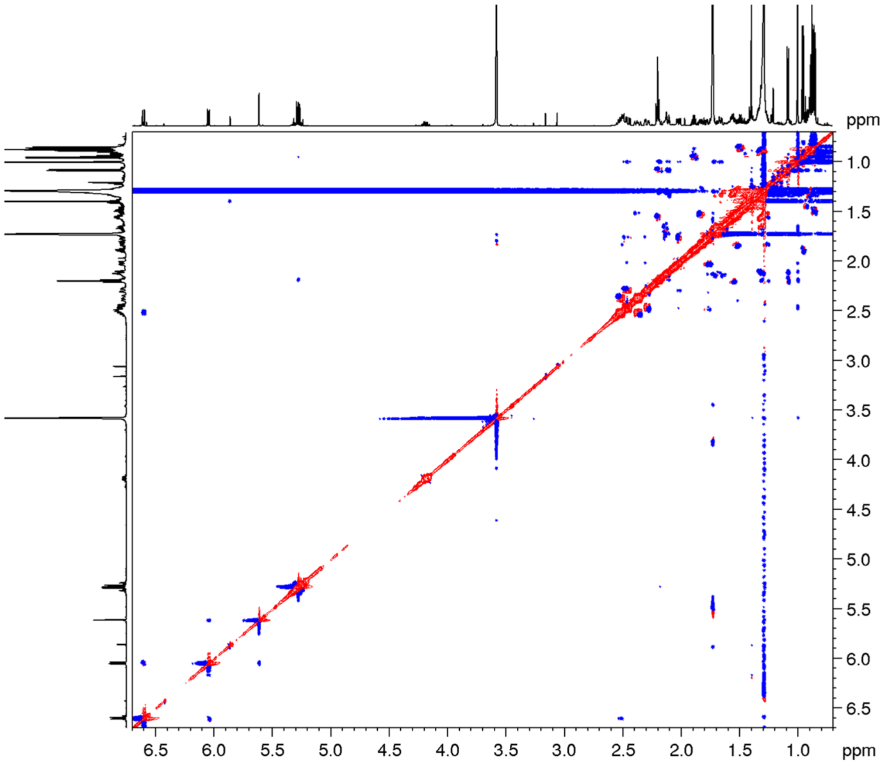

Figure S27. ROESY spectrum of compound **4** (600 MHz, THF-d_8, 295 K)

Spectra and spectral data on compound **5**

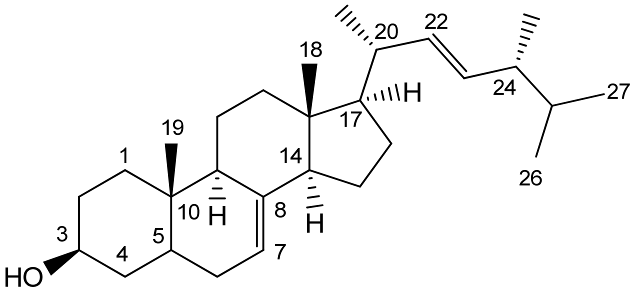

Ergosta-7,22-diene-3-ol (5): amorphous solid; HRESIMS m/z no evaluable data.

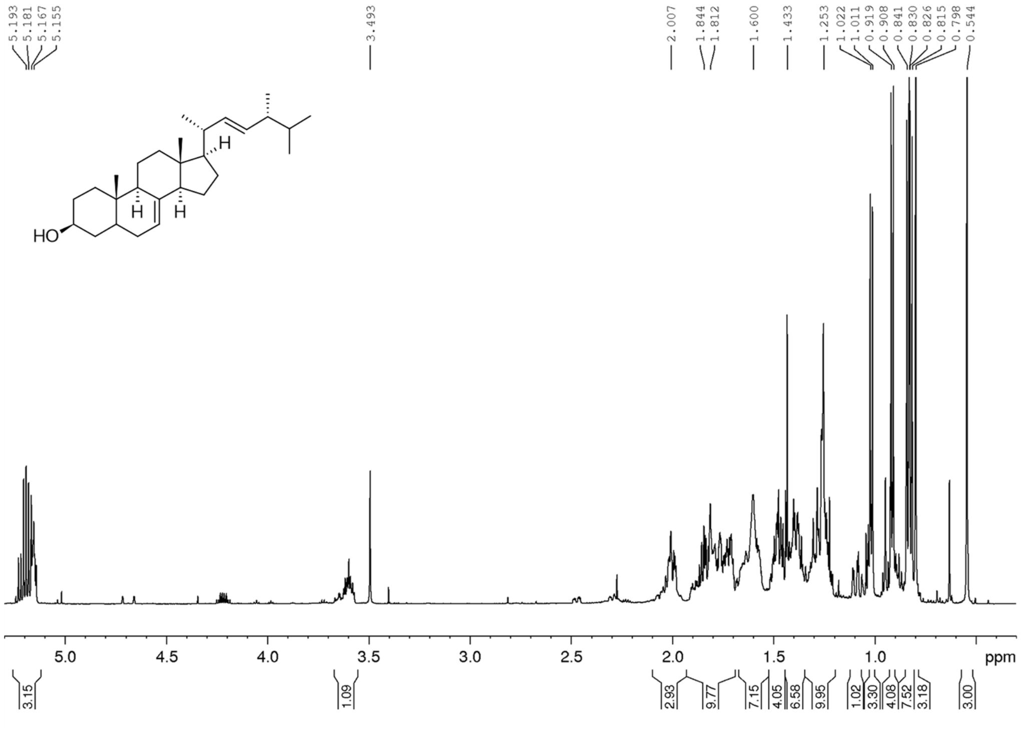

Figure S28. ^{1}H spectrum of compound **5** (600 MHz, $CDCl_3$, 295 K)

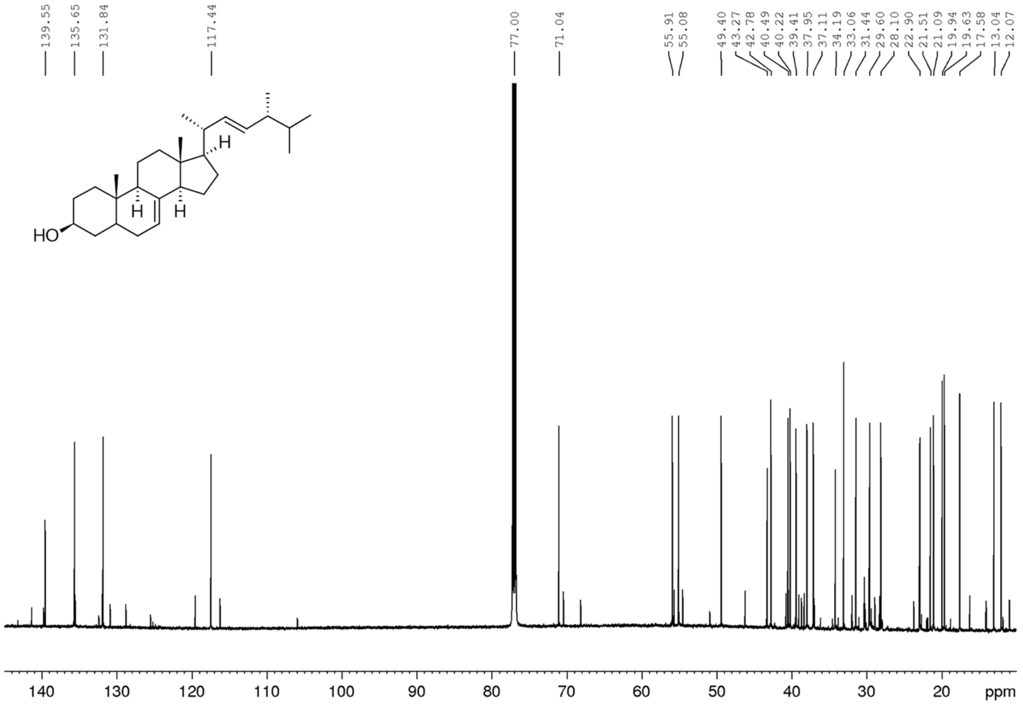

Figure S29. ^{13}C spectrum of compound **5** (150 MHz, $CDCl_3$, 295 K)

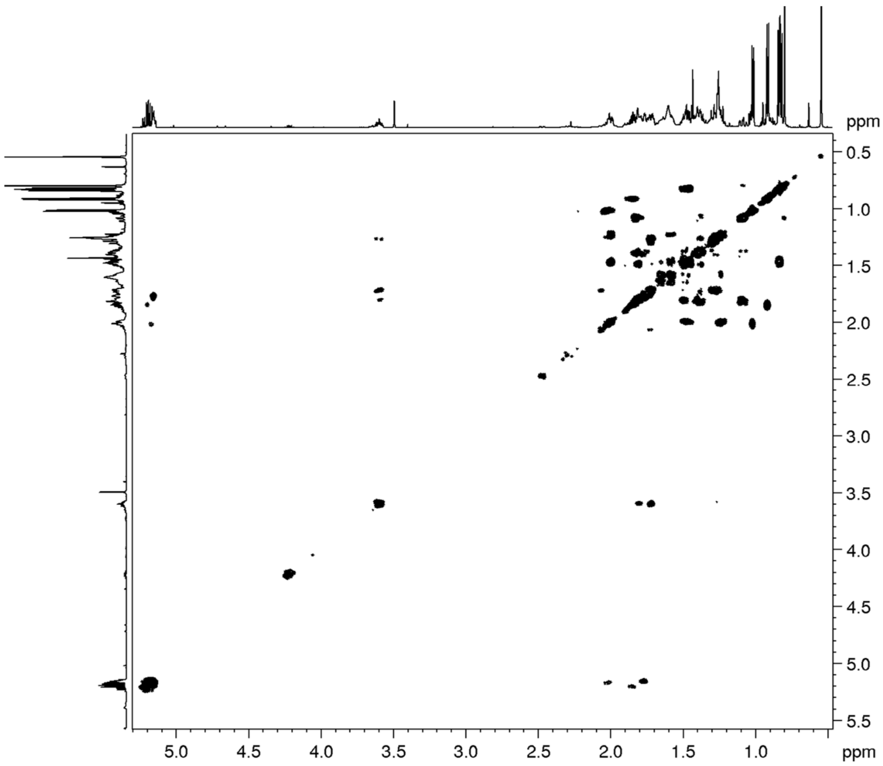

Figure S30. COSY spectrum of compound **5** (600 MHz, $CDCl_3$, 295 K)

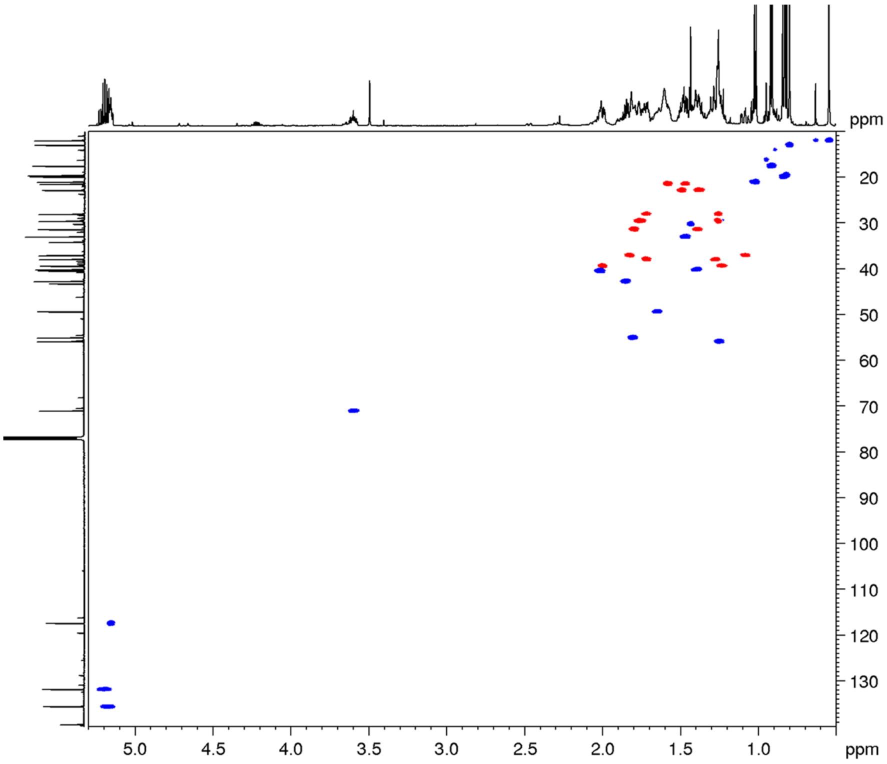

Figure S31. DEPT-edited HSQC spectrum of compound **5** (600 MHz, $CDCl_3$, 295 K)

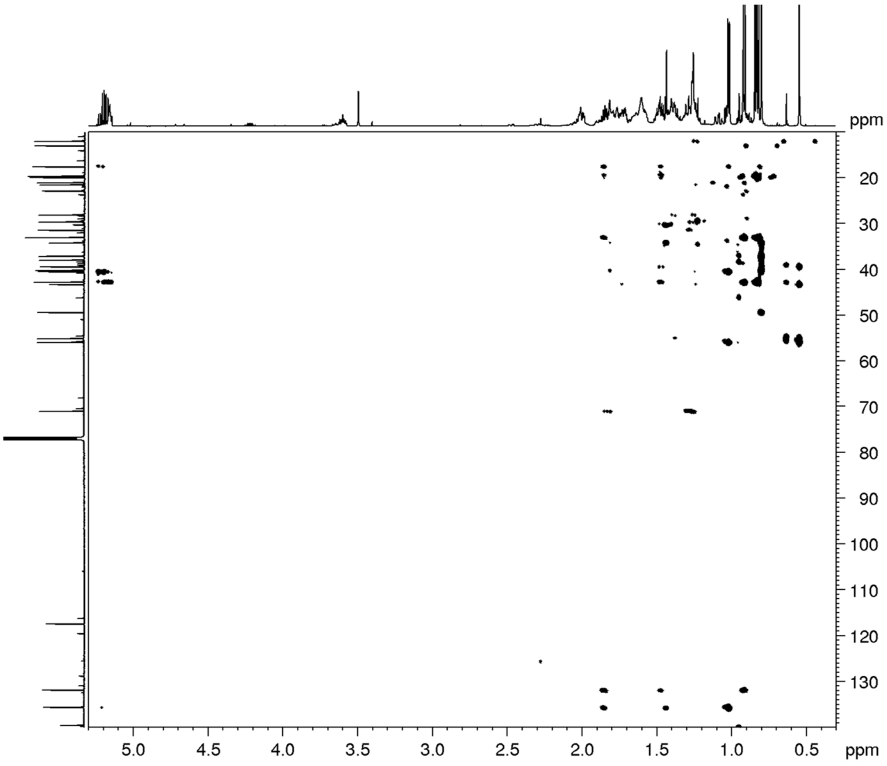

Figure S32. HMBC spectrum of compound **5** (600 MHz, $CDCl_3$, 295 K)

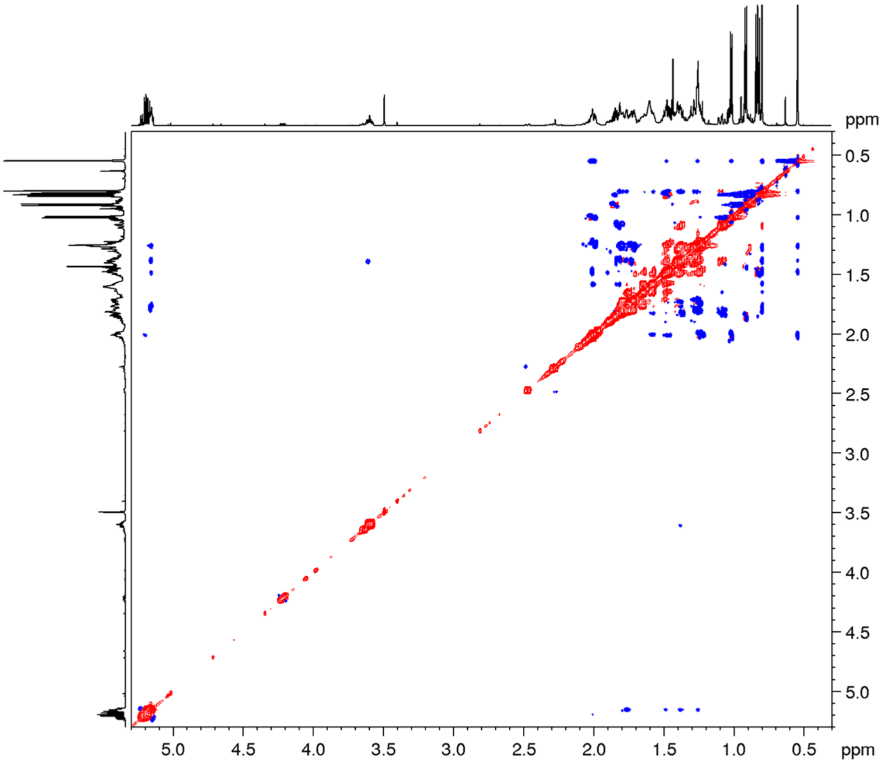

Figure S33. ROESY spectrum of compound **5** (600 MHz, $CDCl_3$, 295 K)

Spectra and spectral data on compound **6**

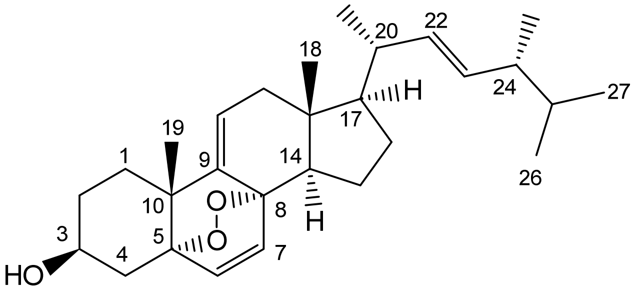

9,11-Dehydroergosterol peroxide (6): amorphous solid; HRESIMS m/z 427.32028 [M + H]$^+$ (Δ 1.1 ppm; $C_{28}H_{43}O_3$); HRESI-MSMS (CID = 15%, 30%, 45%; rel int %) m/z 409(16), 391 (7), 375 (100).

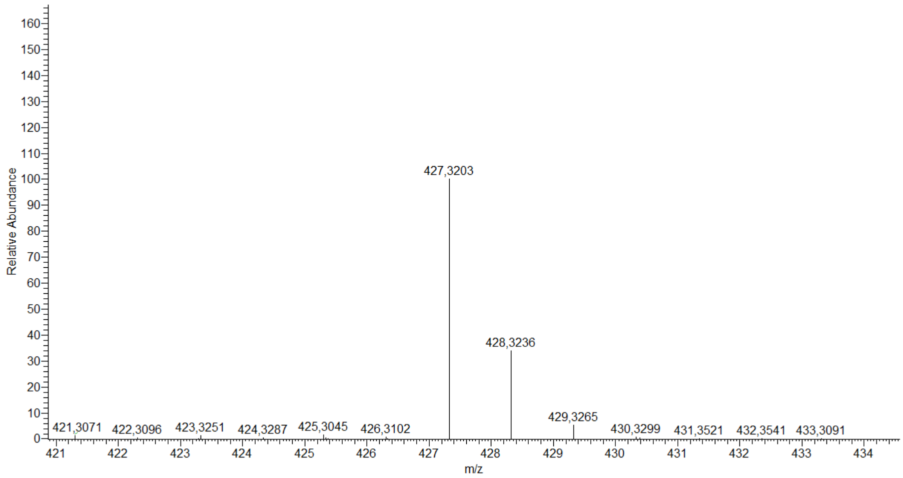

Figure S34. HRESI-MS spectrum of compound **6**

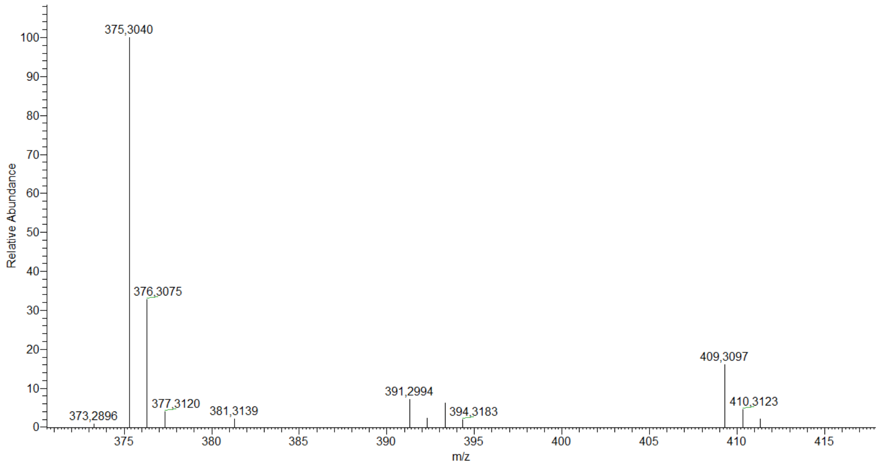

Figure S35. MS-MS spectrum of compound **6**

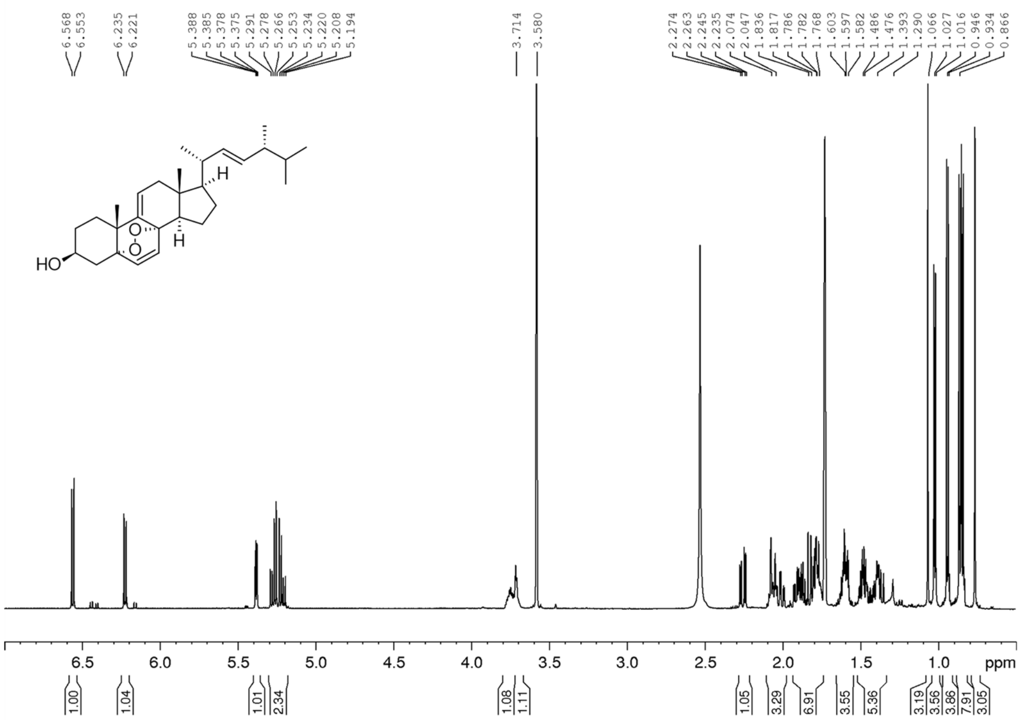

Figure S36. ^{1}H spectrum of compound **6** (600 MHz, THF-d_8, 295 K)

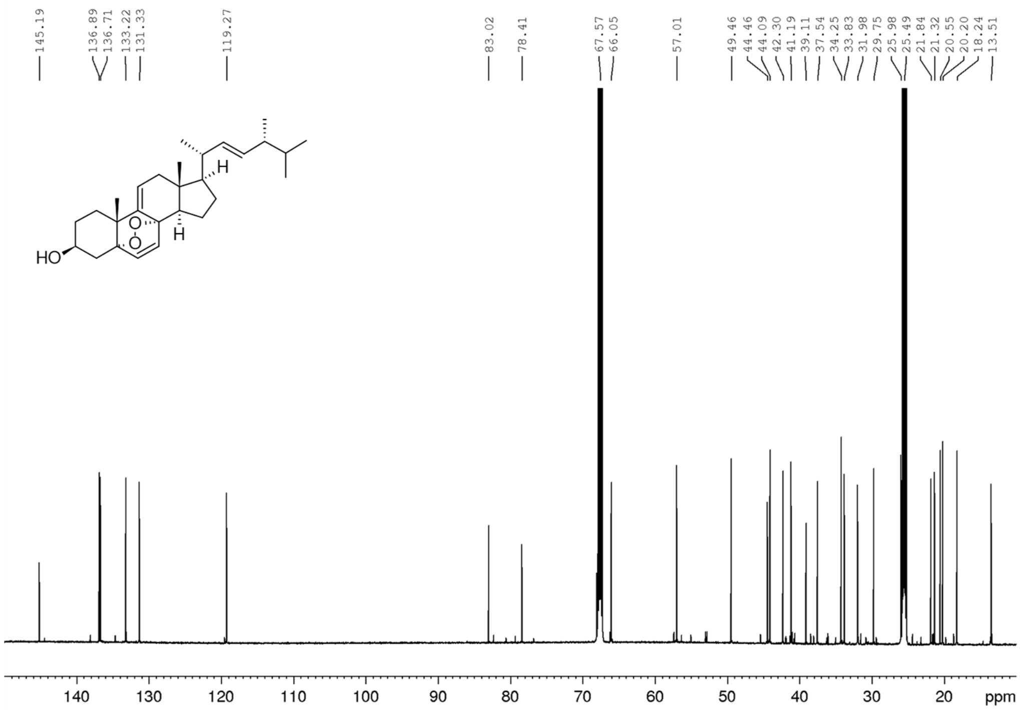

Figure S37. ^{13}C spectrum of compound **6** (150 MHz, THF-d_8, 295 K)

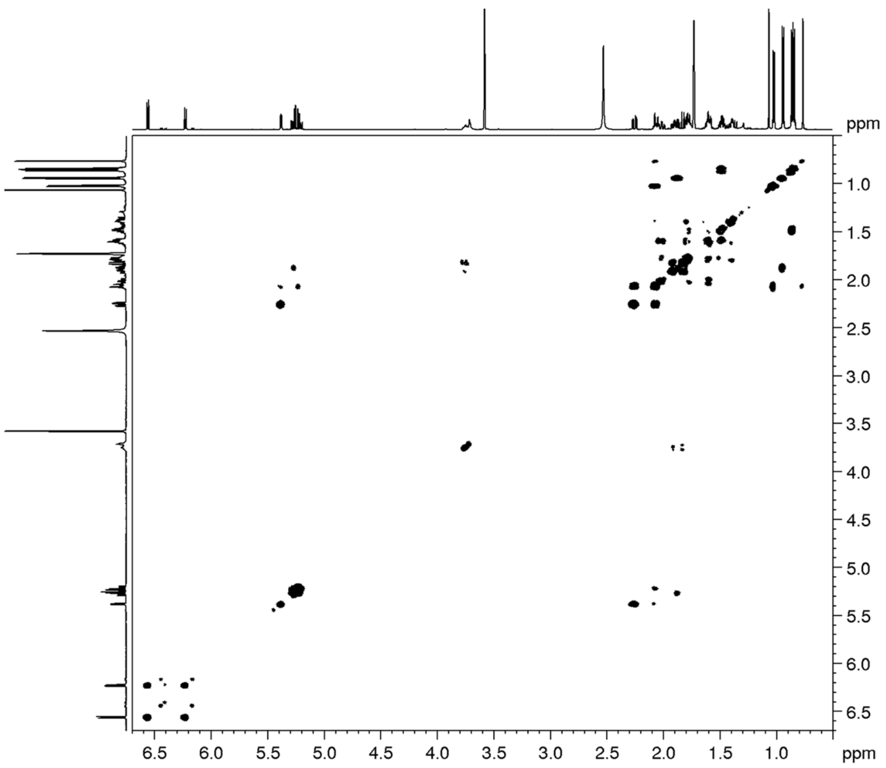

Figure S38. COSY spectrum of compound **6** (600 MHz, THF-d_8, 295 K)

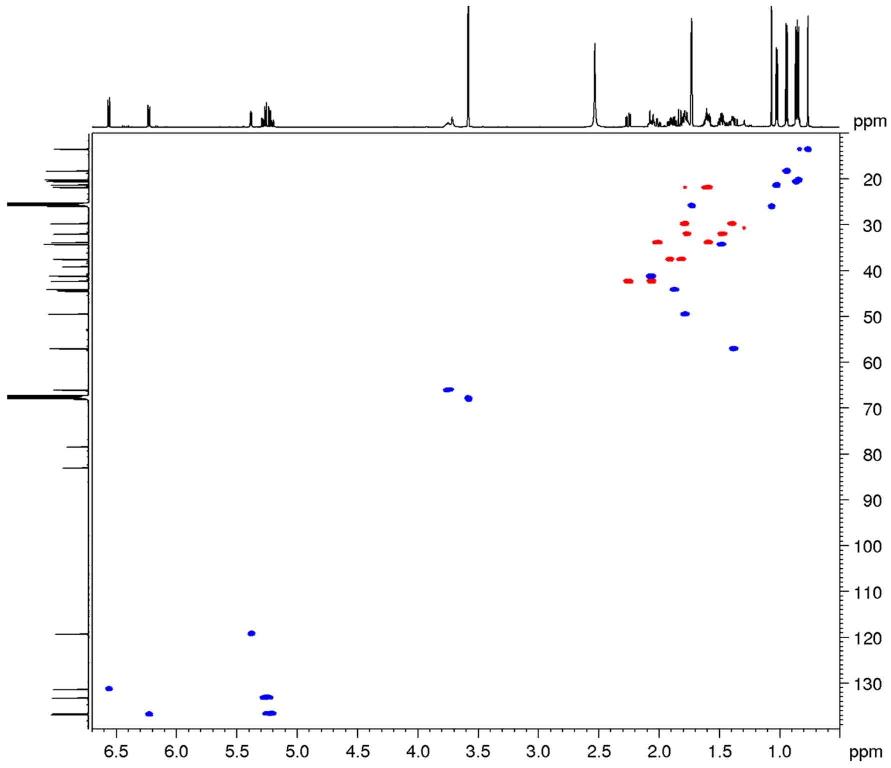

Figure S39. DEPT-edited HSQC spectrum of compound **6** (600 MHz, THF-d_8, 295 K)

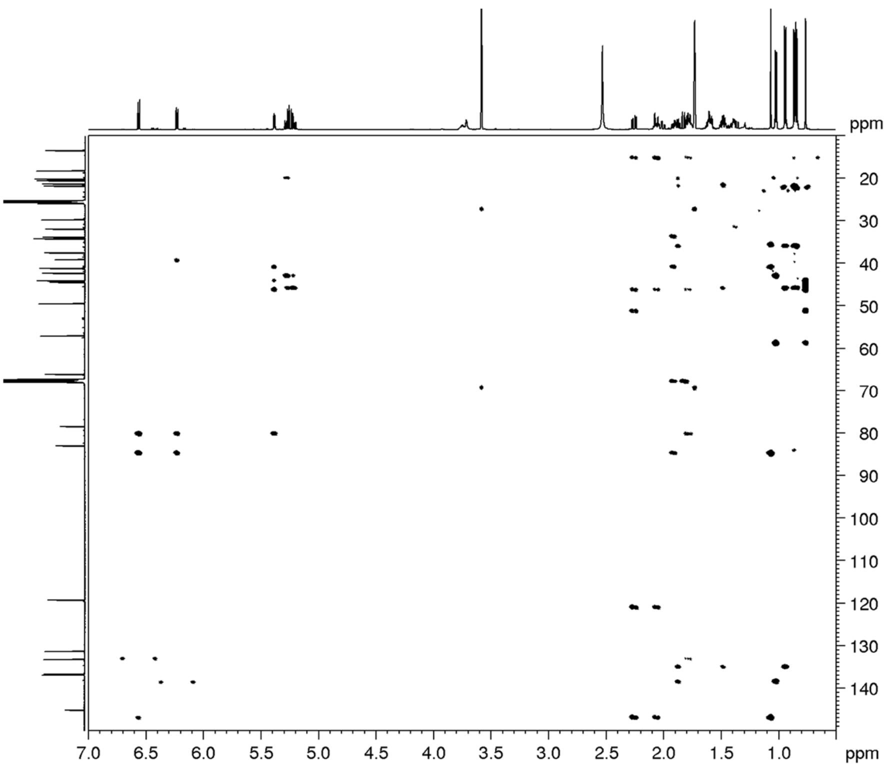

Figure S40. HMBC spectrum of compound **6** (600 MHz, THF-d_8, 295 K)

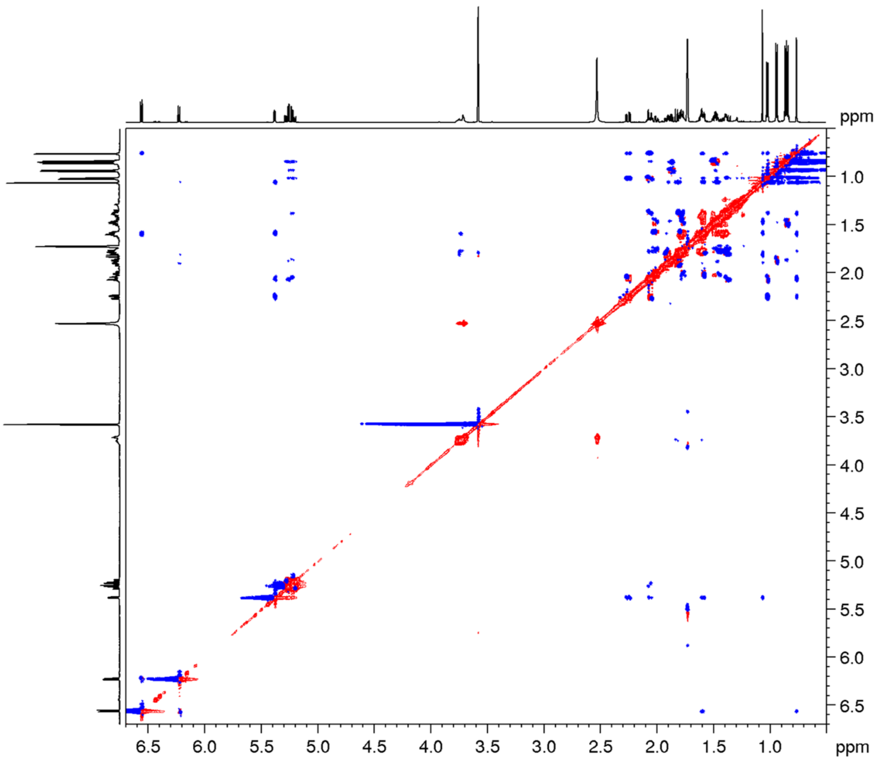

Figure S41. ROESY spectrum of compound **6** (600 MHz, THF-d_8, 295 K)

Spectra and spectral data on compound **7**

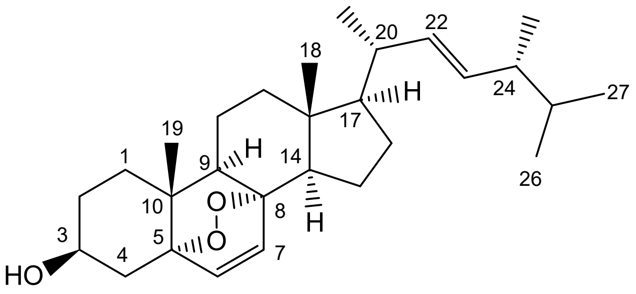

Ergosta-5,7,22-triene-3-ol (7): amorphous solid; HRESIMS m/z 429.33597 $[M + H]^+$ (Δ 0.8 ppm; $C_{28}H_{45}O_3$); HRESI-MSMS (CID = 15%, 30%, 45%; rel int %) m/z 411 (82), 393 (28), 377 (100).

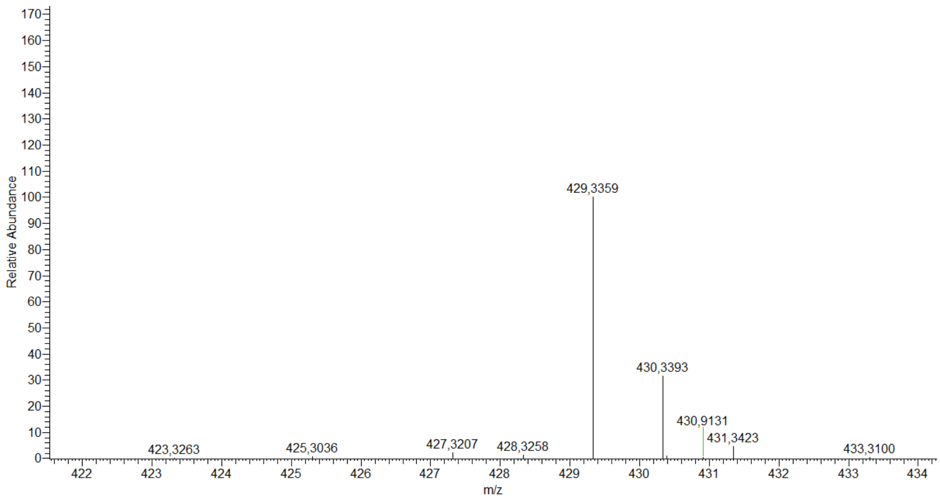

Figure S42. HRESI-MS spectrum of compound **7**

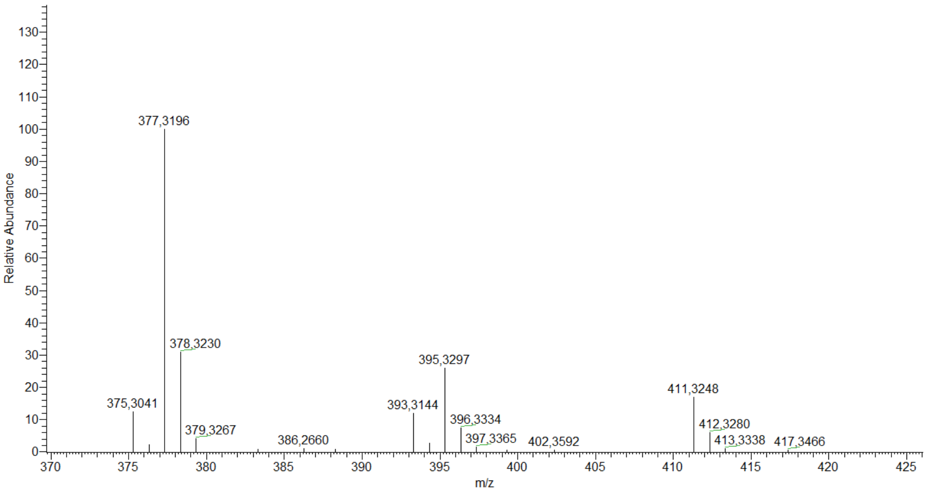

Figure S43. MS-MS spectrum of compound **7**

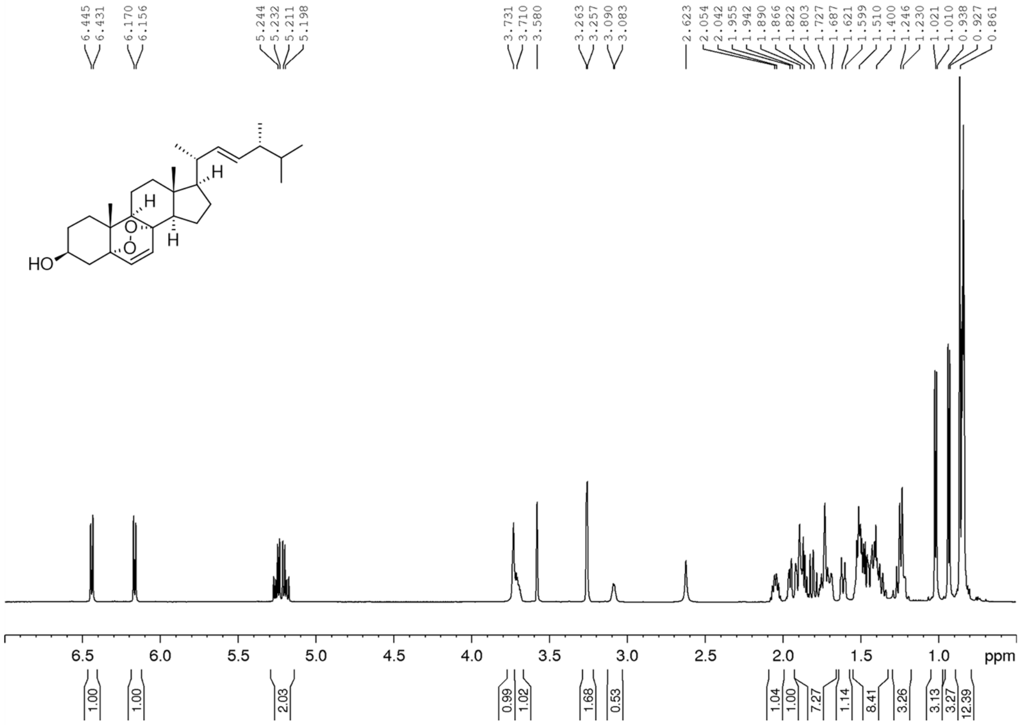

Figure S44. ^{1}H spectrum of compound **7** (600 MHz, THF-d_8, 295 K)

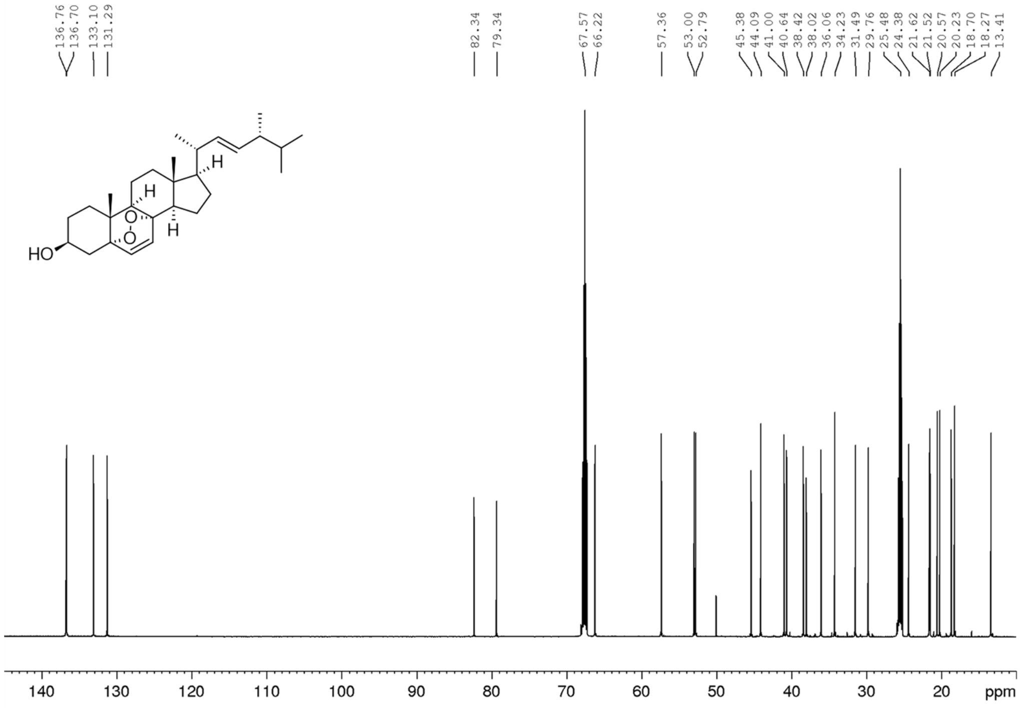

Figure S45. ^{13}C spectrum of compound **7** (150 MHz, THF-d_8, 295 K)

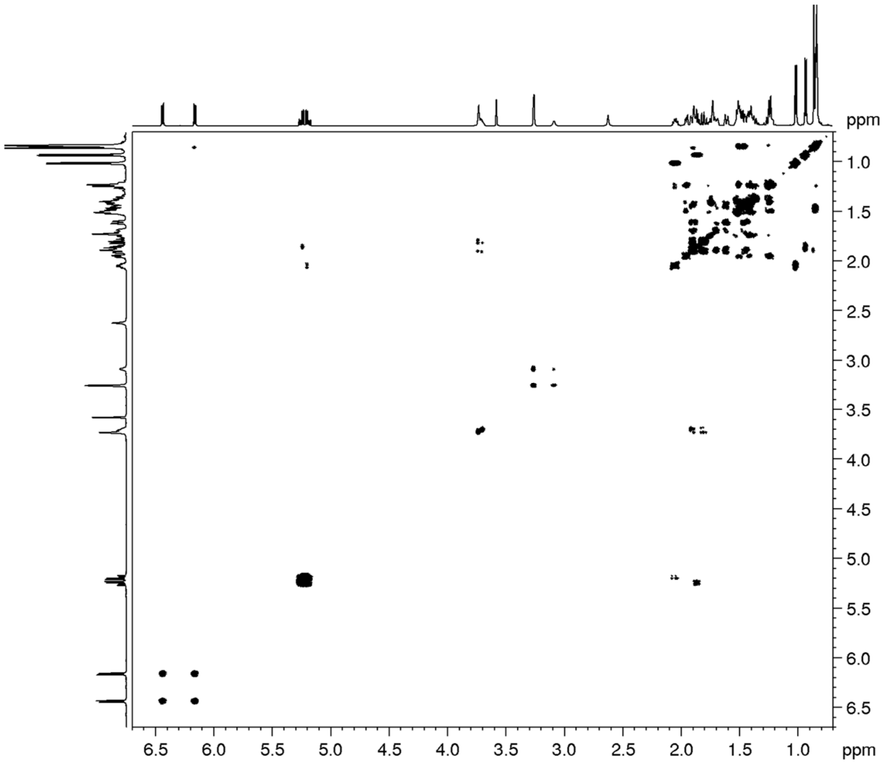

Figure S46. COSY spectrum of compound **7** (600 MHz, THF-d_8, 295 K)

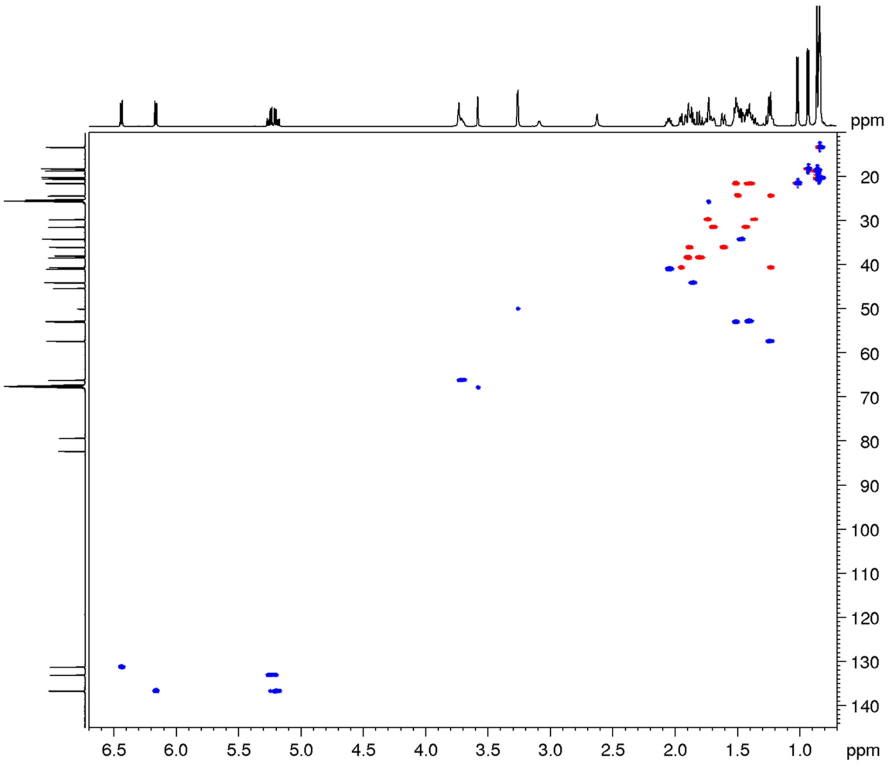

Figure S47. DEPT-edited HSQC spectrum of compound **7** (600 MHz, THF-*d*8, 295 K)

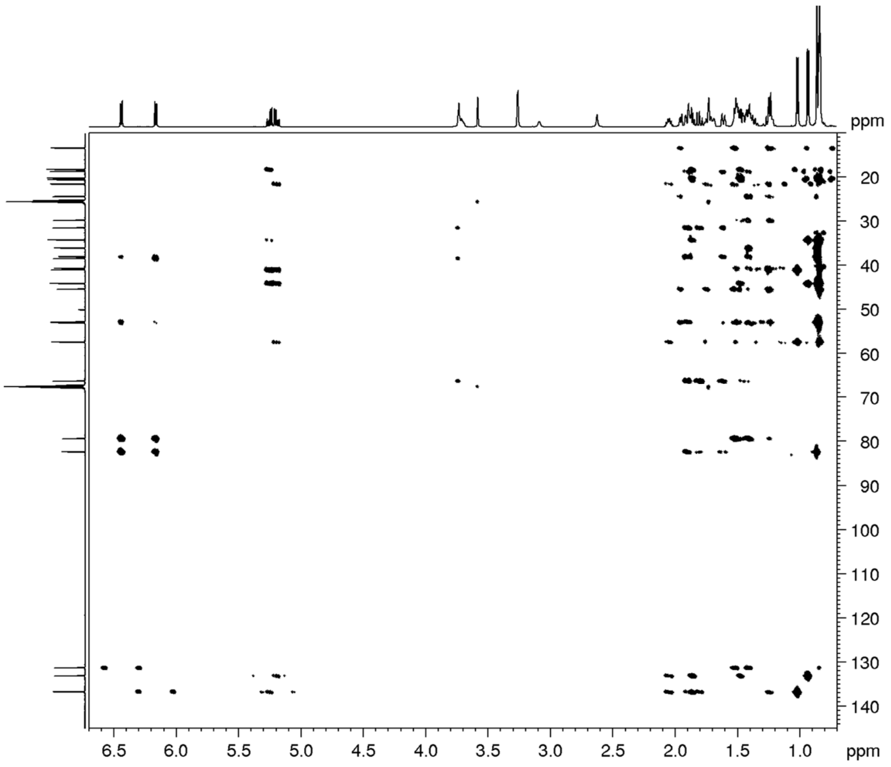

Figure S48. HMBC spectrum of compound **7** (600 MHz, THF-*d*8, 295 K)

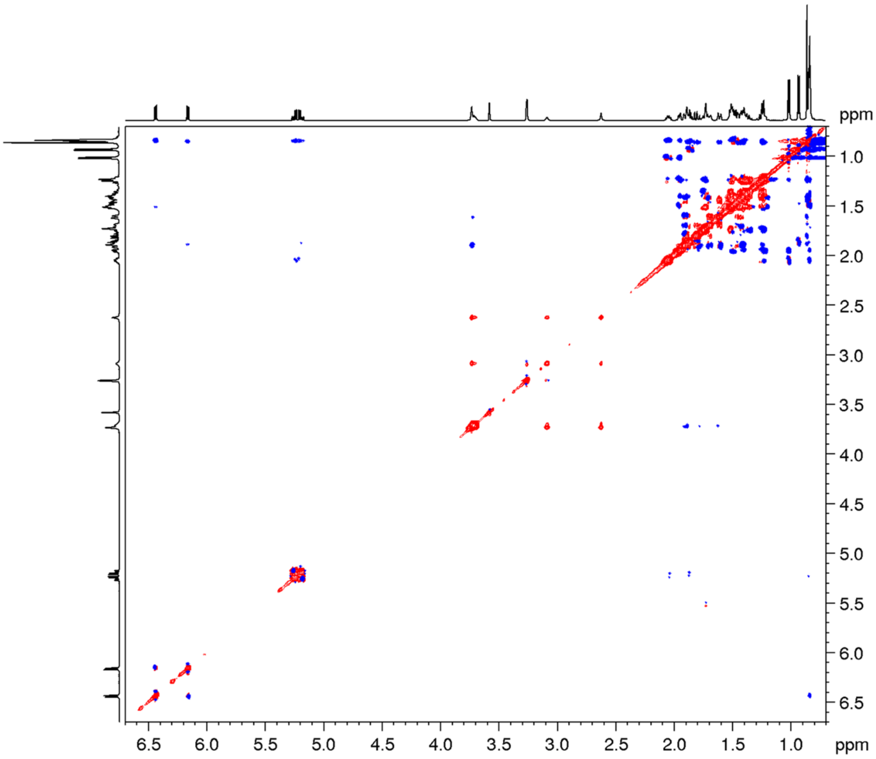

Figure S49. ROESY spectrum of compound **7** (600 MHz, THF-d_8, 295 K)

Table S1. Complete ^{1}H and ^{13}C NMR resonance assignments for compounds **1** and **2**.

No.	**1**			**2**		
	δ ^{13}C	δ ^{1}H	m, *J*	δ ^{13}C	δ ^{1}H	m, *J*
1	33.4	1.73<br>1.38	m<br>m	29.7	1.53<br>1.26	m<br>m
2	25.0	2.09<br>1.62	m<br>m	23.6	1.96<br>1.76	m<br>m
3	76.6	3.93	ddd, 10.1, 6.5, 2.0 Hz	72.5	3.73	b t, 4.6 Hz
4	123.1	5.49	b s	120.9	5.59	d, 4.6 Hz
5	145.5	-	-	147.4	-	-
6	125.8	5.88	d, 9.6 Hz	125.9	5.88	d, 9.7 Hz
7	126.0	6.17	d, 9.6 Hz	126.8	6.21	d, 9.7 Hz
8	124.8	-	-	124.9	-	-
9	45.5	1.97	m	44.9	2.05	m
10	35.9	-	-	36.0	-	-
11	19.1	1.60<br>1.52	m<br>m	19.4	1.64<br>1.52	m<br>m
12	36.3	2.02<br>1.26	m<br>m	36.4	2.00<br>1.25	m<br>m
13	43.5	-	-	43.6	-	-
14	149.7	-	-	149.7	-	-
15	25.0	2.40<br>2.29	m<br>m	25.0	2.40<br>2.28	m<br>m
16	27.9	1.77<br>1.44	m<br>m	27.9	1.77<br>1.44	m<br>m
17	55.9	1.21	m	55.8	1.22	m
18	19.2	0.92	s	19.1	0.92	s
19	18.2	0.89	s	17.1	0.82	s
20	39.4	2.11	m	39.4	2.11	m
21	21.2	1.04	d, 6.7 Hz	21.2	1.04	d, 6.8 Hz
22	135.3	5.21	m	135.4	5.22	m
23	132.2	5.22	m	132.1	5.22	m
24	42.8	1.86	m	42.8	1.87	m
25	33.1	1.48	m	33.1	1.47	m
26	19.7	0.83	d, 6.8 Hz	19.7	0.83	d, 6.8 Hz
27	20.0	0.84	d, 6.8 Hz	20.0	0.84	d, 6.8 Hz
28	17.6	0.92	d, 6.8 Hz	17.6	0.93	d, 6.8 Hz
3-OCH_3	55.4	3.40	s	56.1	3.36	s

Table S2. Complete ^{1}H and ^{13}C NMR resonance assignments for compounds **4**,**5**,**6** and **7**.

**No.**	**4**			**6**			**7**			**5**		
	**δ ^{13}C**	**δ ^{1}H**	**m, *J***	**δ ^{13}C**	**δ ^{1}H**	**m, *J***	**δ ^{13}C**	**δ ^{1}H**	**m, *J***	**δ ^{13}C**	**δ ^{1}H**	**m, *J***
**1**	35.3	2.02<br>1.77	m<br>m	37.1	1.82<br>1.08	m<br>m	33.8	2.01<br>1.59	m<br>m	36.1	1.88<br>1.61	m<br>m
**2**	34.9	2.46<br>2.29	m<br>m	38.0	1.72<br>1.28	m<br>m	32.0	1.76<br>1.47	m<br>m	31.5	1.67<br>1.43	m<br>m
**3**	197.2	-	-	71.0	3.60	m	66.1	3.75	m	66.2	3.71	m
**4**	124.3	5.61	s	31.4	1.80<br>1.39	m<br>m	37.5	1.90<br>1.82	m<br>m	38.4	1.89<br>1.80	m<br>m
**5**	163.3	-	-	40.2	1.40	m	83.0	-	-	82.3	-	-
**6**	125.8	6.05	d, 9.5 Hz	29.6	1.76	m	136.9	6.23	d, 8.5 Hz	136.7	6.16	d, 8.5 Hz
**7**	133.8	6.60	d, 9.5 Hz	117.4	5.16	m	131.3	6.56	d, 8.5 Hz	131.3	6.44	d, 8.5 Hz
**8**	125.9	-	-	139.6	-	-	78.4	-	-	79.3	-	-
**9**	155.7	-	-	49.4	1.65	m	145.2	-	-	52.8	1.40	m
**10**	37.7	-	-	34.2	-	-	39.1	-	-	38.0	-	-
**11**	26.0	2.51<br>2.37	m<br>m	21.5	1.58<br>1.47	m<br>m	119.3	5.38	dd, 6.0. 1.9 Hz	24.4	1.49<br>1.23	m<br>m
**12**	37.0	2.11<br>1.31	m<br>m	39.4	2.00<br>1.23	m<br>m	42.3	2.25<br>2.06	dd, 16.8. 6.0 Hz<br>m	40.6	1.94<br>1.23	m<br>m
**13**	45.0	-	-	43.3	-	-	44.5	-	-	45.4	-	-
**14**	45.7	2.13	m<br>m	55.1<br>22.9	1.81<br>1.49	m<br>m	49.5	1.78	m	53.0	1.51	m
**15**	20.1	1.71<br>1.66	m<br>m	 28.1	1.38<br>1.72	m<br>m	21.8	1.60	m	21.6	1.51<br>1.40	m<br>m
**16**	28.9	1.82<br>1.52	m<br>m	 55.9	1.25<br>1.25	m<br>m	29.8	1.79<br>1.39	m<br>m	29.8	1.73<br>1.37	m<br>m
**17**	57.0	1.27	m	12.1	0.54	s	57.0	1.38	m	57.4	1.24	m

**18**	19.4	1.00	s	13.0	0.80	s	13.5	0.76	s	13.4	0.83	m
**19**	17.1	1.00	s	40.5	2.01	m	26.0	1.07	s	18.7	0.86	m
**20**	40.6	2.19	m	21.1	1.02	d, 6.8 Hz	41.2	2.07	m	41.0	2.04	m
**21**	21.8	1.08	d, 6.7 Hz	135.7	5.17	m	21.3	1.02	d, 6.7 Hz	21.5	1.02	d, 6.5 Hz
**22**	136.5	5.27	m	131.8	5.20	m	136.7	5.23	m	136.8	5.20	m
**23**	133.4	5.28	m	42.8	1.85	m	133.2	5.26	m	133.1	5.23	m
**24**	44.2	1.89	m	33.1	1.47	m	44.1	1.87	m	44.1	1.86	m
**25**	34.3	1.48	m	19.6	0.82	d, 6.8 Hz	34.3	1.48	m	34.2	1.47	m
**26**	20.2	0.85	d, 6.8 Hz	19.9	0.84	d, 6.8 Hz	20.2	0.84	d, 6.8 Hz	20.2	0.83	m
**27**	20.6	0.87	d, 6.8 Hz	17.6	0.91	d, 6.8 Hz	20.6	0.86	d, 6.8 Hz	20.6	0.85	m
**28**	18.3	0.96	d, 6.8 Hz				18.2	0.94	d, 6.8 Hz	18.3	0.93	m

Table S3. Complete ^1H and ^{13}C NMR resonance assignments for compound **3**

**No.**	**3**		
	**δ ^{13}C**	**δ ^1H**	**m, *J***
**2**	87.2	5.30	dd, 9.7; 7.9 Hz, 1H
**3**	33.7	3.41	dd, 15.9; 9.7 Hz, 1H
		3.09	dd, 15.9; 7.9 Hz, 1H
**3a**	128.2	-	-
**4**	125.8	7.73	M
**5**	130.4	-	-
**6**	133.2	7.68	dd, 8.2; 1.7 Hz
**7**	109.5	6.90	d, 8.2 Hz
**7a**	165.2	-	-
**1′**	143.1	-	-
**2′**	112.8	5.10	m
		4.95	m
**3′**	17.0	1.77	s
**1″**	190.7	9.83	s
